# Supplementary material for: Hippocampal Neurogenesis Requires Cell-Autonomous Thyroid Hormone Signaling
Source: Stem Cell Reports. 2020 Apr 16;14(5):845–60. doi: 10.1016/j.stemcr.2020.03.014 (PMC7220957; doi:10.1016/j.stemcr.2020.03.014)
Supplement: Document S1. Supplemental Experimental Procedures and Figures S1–S7 [file mmc1.pdf]

Stem Cell Reports, Volume *14*

Supplemental Information

Hippocampal Neurogenesis Requires Cell-Autonomous Thyroid Hormone Signaling

Steffen Mayerl, Heike Heuer, and Charles ffrench-Constant

Fig.S1

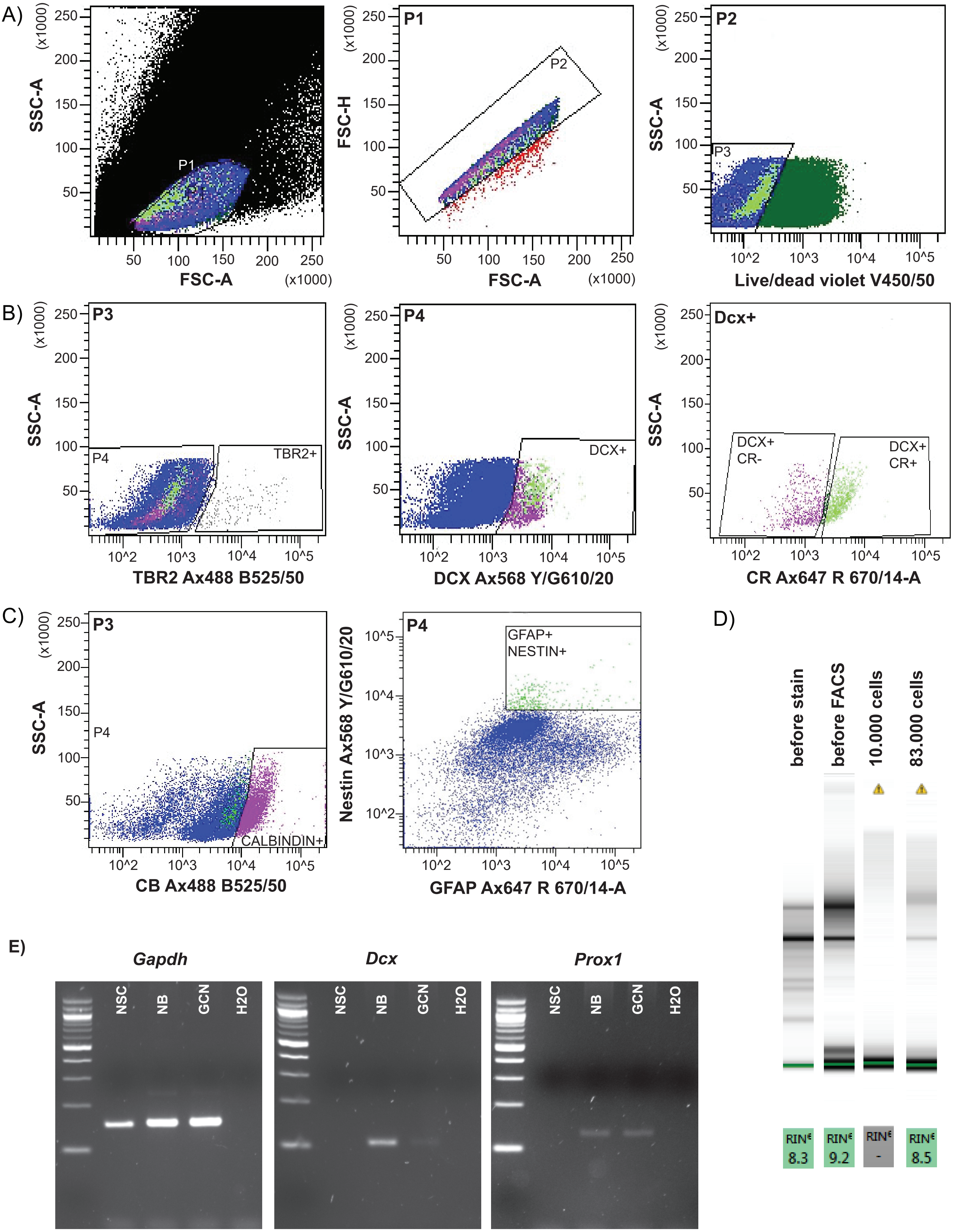
A)
P1
P2
P3
SSC-A
FSC-H
FSC-A
(x1000)
Live/dead violet V450/50
B)
P3
P4
TBR2+
DCX+
Dcx+
DCX+ CR-
DCX+ CR+
TBR2 Ax488 B525/50
DCX Ax568 Y/G610/20
CR Ax647 R 670/14-A
C)
CALBINDIN+
GFAP+ NESTIN+
Nestin Ax568 Y/G610/20
CB Ax488 B525/50
GFAP Ax647 R 670/14-A
D)
before stain
before FACS
10.000 cells
83.000 cells
RIN 8.3
RIN 9.2
RIN -
RIN 8.5
E)
Gapdh
Dcx
Prox1
NSC
NB
GCN
H2O

Fig.S2

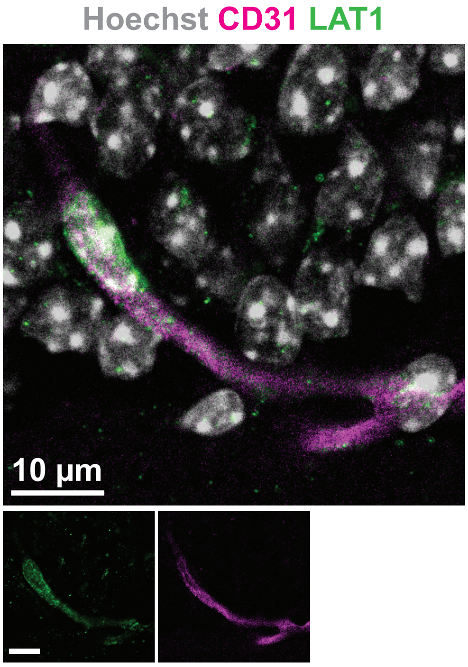

Fig.S3

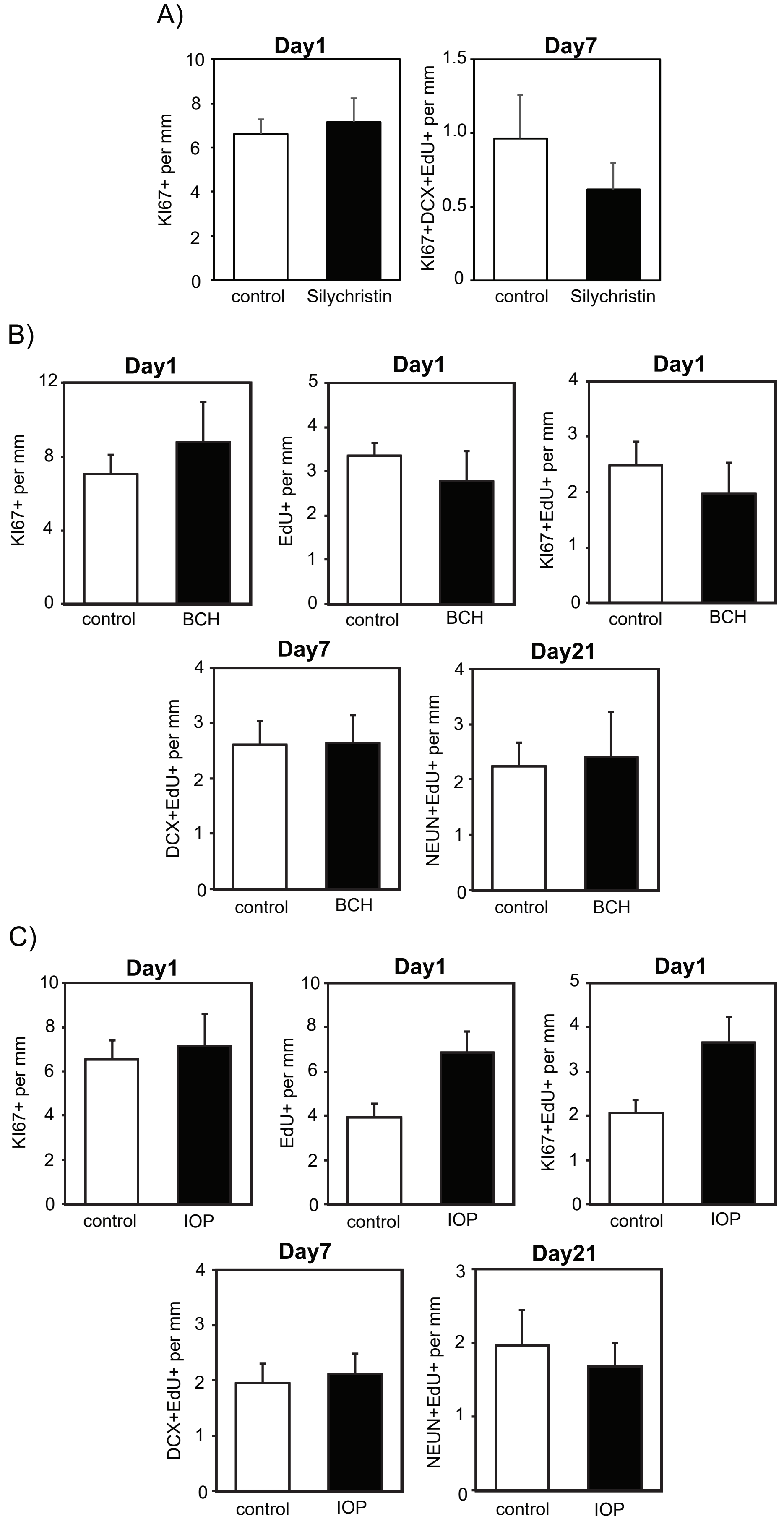
A)
Day1
KI67+ per mm
control
Silychristin
Day7
KI67+DCX+EdU+ per mm
control
Silychristin
B)
Day1
KI67+ per mm
control
BCH
Day1
EdU+ per mm
control
BCH
Day1
KI67+EdU+ per mm
control
BCH
Day7
DCX+EdU+ per mm
control
BCH
Day21
NEUN+EdU+ per mm
control
BCH
C)
Day1
KI67+ per mm
control
IOP
Day1
EdU+ per mm
control
IOP
Day1
KI67+EdU+ per mm
control
IOP
Day7
DCX+EdU+ per mm
control
IOP
Day21
NEUN+EdU+ per mm
control
IOP

Fig.S4

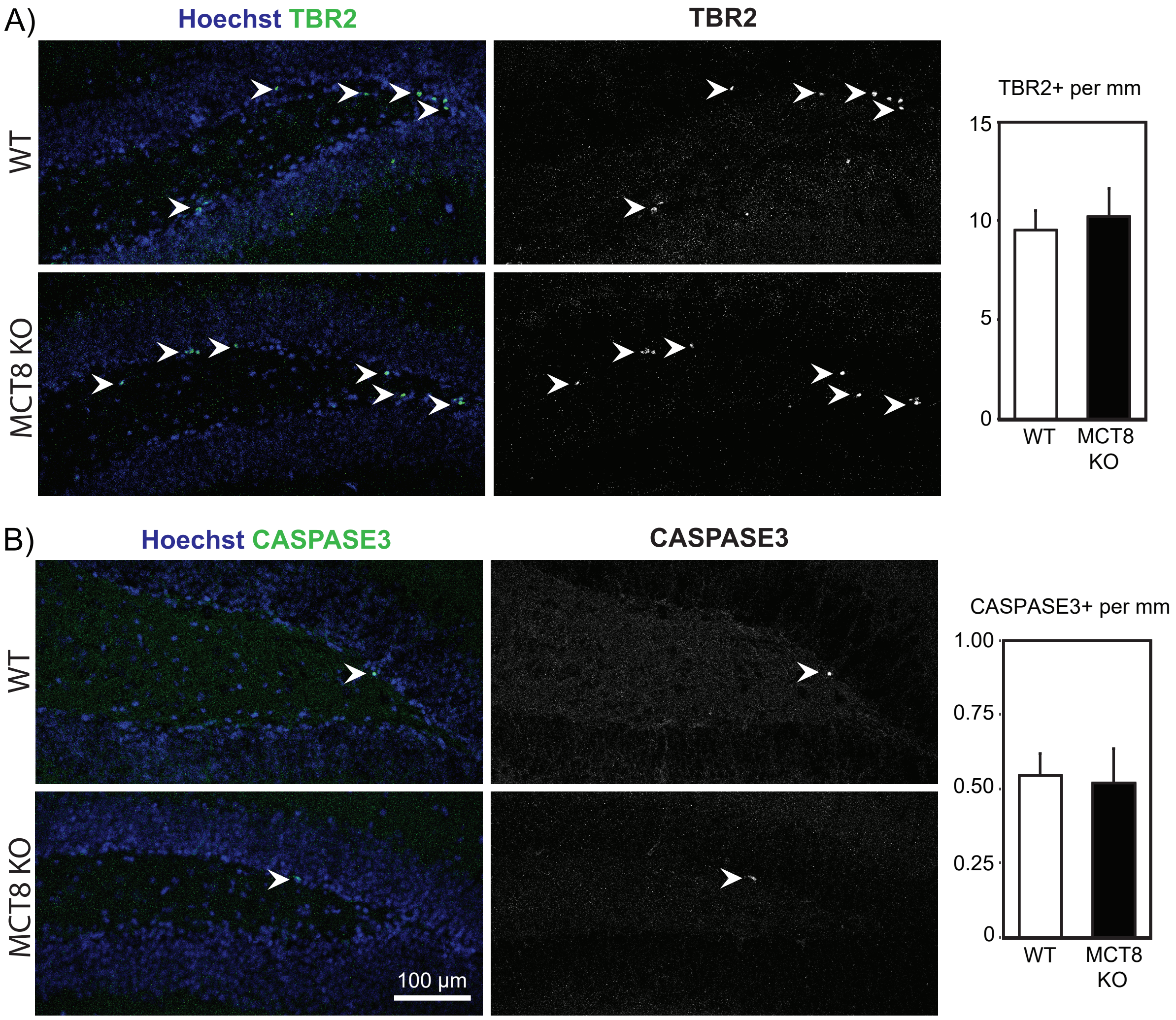
A)
Hoechst TBR2
TBR2
WT
MCT8 KO
TBR2+ per mm
15
10
5
0
WT
MCT8 KO
B)
Hoechst CASPASE3
CASPASE3
WT
MCT8 KO
100 µm
CASPASE3+ per mm
1.00
0.75
0.50
0.25
0
WT
MCT8 KO

Fig.S5

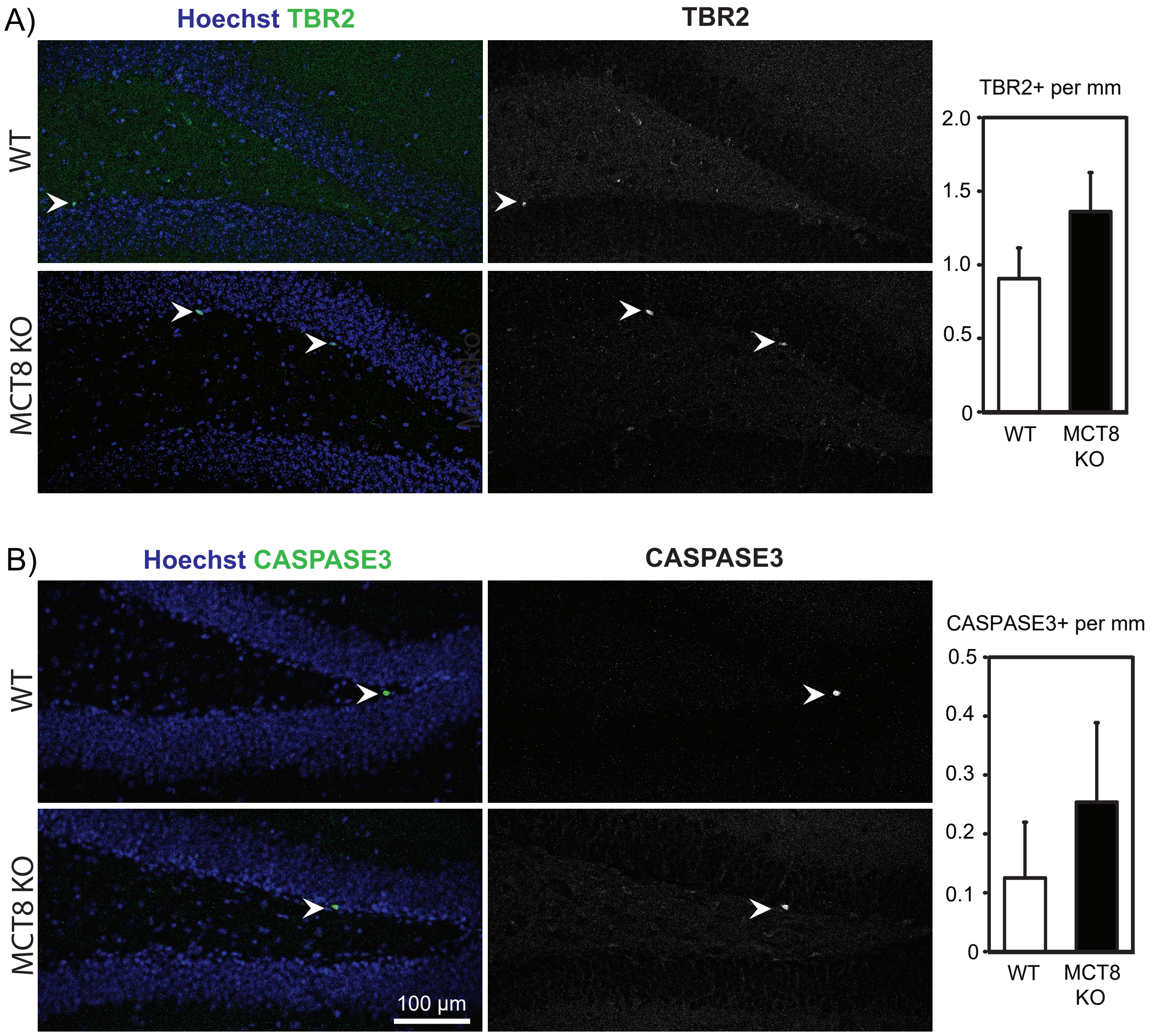
A)
Hoechst TBR2
TBR2
WT
MCT8 KO
TBR2+ per mm
2.0
1.5
1.0
0.5
0
WT
MCT8 KO
B)
Hoechst CASPASE3
CASPASE3
WT
MCT8 KO
100 µm
CASPASE3+ per mm
0.5
0.4
0.3
0.2
0.1
0
WT
MCT8 KO

Fig.S6

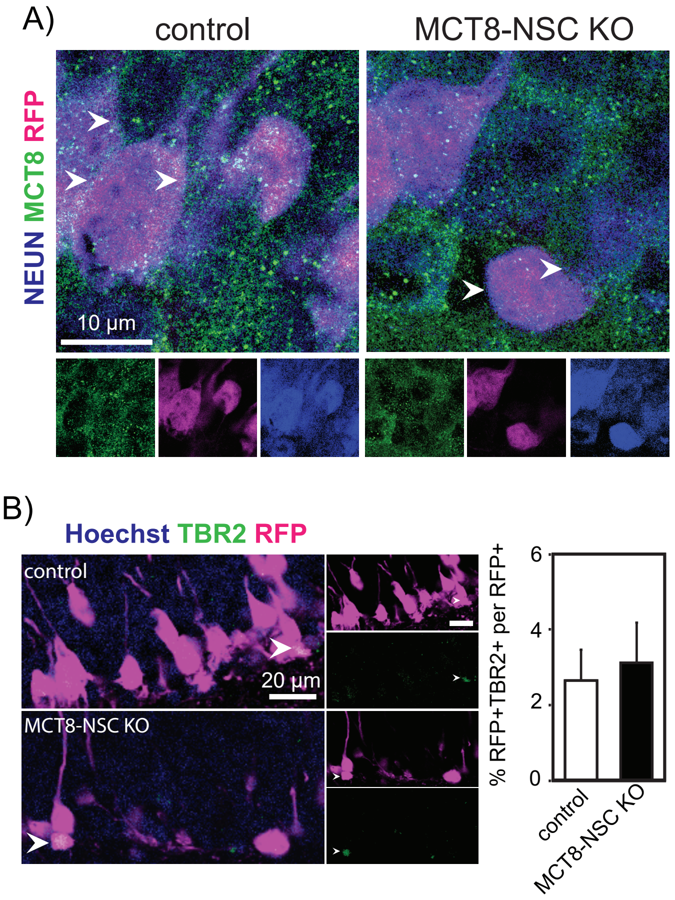
A)
control
MCT8-NSC KO
NEUN MCT8 RFP
10 µm
B)
Hoechst TBR2 RFP
control
20 µm
MCT8-NSC KO
% RFP+TBR2+ per RFP+
6
4
2
0
control
MCT8-NSC KO

Fig.S7

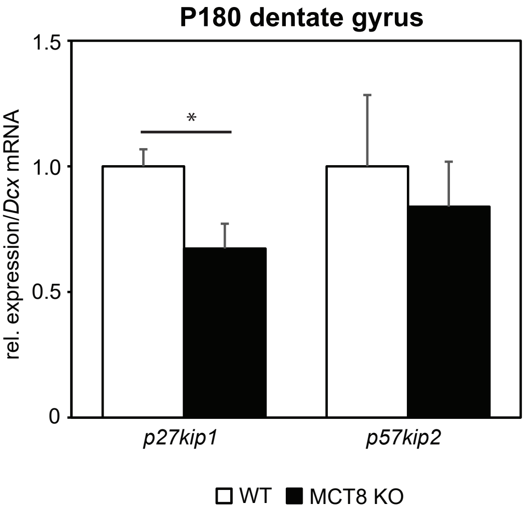
P180 dentate gyrus
1.5
1.0
0.5
0
rel. expression/Dcx mRNA
*
p27kip1
p57kip2
WT
MCT8 KO

Supplementary figure legends:

Fig.S1: FACsorting strategy and RNA integrity control. Related to Fig. 1

Micro-dissected dentate gyri were subjected to flow cytometry and different neurogenic/neuronal populations were sorted according to their expression of intracellular markers. A) Flow cytometry strategy is shown to separate cells from debris (P1), single cells from cell clusters (P2) and living cells within the P2 population that were identified by low levels of a fixable live/dead cell stain (P3). B) Samples were stained for TBR2(AxF488), DCX(AxF568) and CR(AxF647) and TBR2+ (TAP), DCX+/CR- (NB) as well as DCX+/CR+ (IN) cell populations were separated. TBR2-/DCX- cells were collected as control for RIN value determination. C) Samples were stained for CB(AxF488), NESTIN(AxF568) and GFAP(AxF647). First, CB+ cells were separated (GCN) followed by sorting into NESTIN+/GFAP+ (NSC) and non-NSCs used for RIN value determination. D) RIN values were assessed after indicated steps. After FACS, higher cell numbers (≥83.000) were needed to determine reliably RIN values. E) RNA was isolated, amplified and reversely transcribed from different samples and subjected to RT-PCR. Expression pattern of the house keeping gene *Gapdh* and neurogenic markers *Dcx* and *Prox1* were evaluated. NSC-neural stem cells; TAP-transiently amplifying progenitors; NB-neuroblasts; IN-immature neurons; GCN-granule cell neurons.

Fig.S2: Expression of TH signaling components in the DG. Related to Fig. 2

Coronal forebrain cryosections were incubated with antibodies against LAT1 (in green) and the endothelial cell marker CD31/PECAM (in magenta). Nuclei stained with Hoechst33258 appear gray.

Fig.S3: BCH and IOP do not alter hippocampal neurogenesis ex vivo. Related to Fig. 3

Adult mouse brain slices were kept in culture for up to 21 days in the presence of inhibitors of TH signaling components. A) Number of proliferating cells (KI67+) in the SGZ after 1 day and

number of proliferating NBs (KI67+/DCX+/EdU+) after 7 days in the presence of the MCT8 inhibitor Silychristin were determined. B) Slices were exposed to BCH, an inhibitor of L-type amino acid transporters 1/2, and overall proliferation (KI67+ cell numbers), EdU incorporation in general and into proliferating cells after 1 day in culture; in EdU/DCX co-labelled cells after 7 days in culture or in EdU/NEUN double positive cells after 21 days in culture was quantified. C) Slices were cultured in the presence of the deiodinase inhibitor iopanoic acid (IOP). Proliferation at day 1 as well as EdU-incorporation into KI67+ cells at day 1, into DCX+ progenitors at day 7 and into neurons at day 21 is depicted. n=4-6 mice per condition. Group means + SEM are shown.

Fig.S4: Progenitors in the SGZ at two months of age. Related to Fig. 4

Intermediate progenitors and apoptosis were analyzed on perfusion-fixed forebrain cryosections of WT and MCT8 KO mice at 2 months of age. A) Number of cells positive for the intermediate progenitor marker TBR2 (in green) were enumerated. B) Number of apoptotic cells (cleaved caspase-3 positive; green) was compared between WT and MCT8 KO littermates. Hoechst33258 counter-stained nuclei appear blue. n=6 mice per genotype. Group means + SEM are shown.

Fig.S5: Progenitors in the SGZ at six months of age. Related to Fig. 5

At 6 months of age, intermediate progenitor numbers positive for TBR2 (in green/grey; arrowheads) (A) and cleaved caspase-3 positive (in green/grey; arrowheads) apoptotic cells (B) were compared between WT and MCT8 KO mice using perfusion-fixed forebrain cryosections. Hoechst33258 counter-stained nuclei appear blue. n=6 mice per genotype. Group means + SEM are shown.

Fig.S6: Intermediate progenitors in MCT8-NSC KO animals. Related to Fig. 6

Conditional deletion of MCT8 in adult NSCs was achieved by Tamoxifen-induced activation of a *Nestin*-CreERT2 construct at 4 weeks of age and monitored by RFP reporter. A) Adult

generated neurons positive for NEUN (blue) and RFP (magenta) express MCT8 (green; arrows) in control animals, but are devoid of specific MCT8 staining in the MCT8-NSC KO condition when analyzed at 6 months of age. B) In 6 months old animals, relative numbers of TBR2+ (green)/RFP+ (magenta) TAPs in the dentate gyrus were determined and are presented as % of all RFP+ cells. Cell nuclei are labelled with Hoechst33258 and are shown in blue. n=5 mice per genotype. Group means + SEM are shown.

Fig.S7: Cell cycle inhibitor transcript levels in the dentate gyrus. Related to Fig. 7
At 6 months of age, dentate gyri were micro-dissected and cDNA was generated. Transcript expression of *p27kip1* and *p57kip2* was investigated and levels were normalized to that of the stage-specific marker *Dcx*. n=4-5 mice per genotype. Group means + SEM are shown. *, $p < 0.05$, unpaired two-tailed Student's *t* test.

Supplementary experimental procedures:

Tissue preparation for FACS

Dentate gyri were minced with a razor blade on an ice-cold glass plate, collected into Hibernate A and centrifuged for 2 min at 3000 rpm. Generally, all centrifugation steps were conducted at 4 C. Hibernate A was replaced with 1 ml Accutase (Millipore) and tissue was digested for 30 min at 4 C. Subsequently, tissue was pelleted by centrifugation for 2 min at 3000 rpm and re-suspended in 1 ml Hibernate A. To dissociate cells, dentate gyri pieces were triturated on ice using a syringe and needles with decreasing diameter (1.2 mm, 0.8 mm and 0.4 mm). Cells were filtered through a pre-wetted 40 µm cell strainer into a 50 ml tube to remove large debris. Smaller debris was reduced by applying cell solution on top of a three-step Percoll gradient (for recipe see (Guez-Barber et al., 2012)). After initial centrifugation at 4000 rpm for 4 min, the cloudy top layer was aspirated and cells were pelleted by centrifugation at 5000 rpm for 10 min.

Staining procedure for FACS

Following re-suspension in 860 µl ice-cold staining buffer (1x PBS with 0.1% saponine, 1% BSA and 1:25 RNaseOUT) three 20 µl aliquots were taken (for an unstained control and two secondary only controls) and the remaining volume was split into two 400 µl aliquots. The first aliquot was incubated with mouse anti-CR (1:400; Swant #6B3), guinea pig anti-DCX (1:400; Merck Millipore #AB2253) and rabbit anti-TBR2 (1:150; Abcam #ab183991); the second 400 µl cell suspension was incubated with rabbit anti-CB (1:100; Millipore #AB1778), mouse anti-GFAP (1:200;Merck Millipore #MAB360) and chicken anti-NESTIN (1:150; Aves #NES) for 40 min at 4 C. Cells were washed, re-suspended in 400 µl ice-cold staining buffer and incubated with Alexa 488/568/647-coupled secondary antibodies raised in goat (1:400; Invitrogen) for 30 min at 4 C. For secondary only controls, 380 µl staining buffer were added to a 20 µl cell aliquot and cells were incubated with 0.5 µl of a 1:10 mix of all secondary antibodies in staining buffer. After washing, 400 µl ice-cold FACS buffer was added (1x PBS containing 0.5% BSA and 1:50 RNaseOUT) per sample.

Immunofluorescence studies

Coronal forebrain cryosections containing the hippocampus (16 µm) were thaw-mounted on superfrost slides (Thermo Scientific), post-fixed with 4% PFA for 10 min and permeabilized with 0.1% Triton X-100/0.1 M glycine. For EdU detection, sections were blocked in 3% BSA in PBS and Click-iT reaction was performed using the Click-iT® EdU Alexa Fluor® 647 Imaging Kit (Thermo Fisher Scientific) according to the manufacturer’s instructions. Subsequently, sections were blocked with MOM reagent (1:40; Vector laboratories) in blocking buffer (PBS containing 10% goat serum and 0.2% Triton X-100) and incubated with the respective primary antibodies in blocking buffer overnight at 4 C. Sections were incubated with Alexa Fluor 488, 568 or 647 labelled secondary antibodies (all 1:1000) in blocking buffer and Hoechst33258 (5 µg/ml), mounted with Fluoromount-G* (Southern Biotech) and analysed using a Leica SP8 confocal microscope.

Organotypic adult hippocampal slices were permeabilized and subjected to Click-iT reaction as above with extended incubation times (2 h blocking in 3% BSA/PBS and 3 h incubation with the Click-iT reaction cocktail). Free-floating slices were blocked overnight in blocking buffer (with 0.5% Triton X-100), incubated for 48 h with primary antibodies and 24 h with secondary antibodies as above in blocking buffer (with 0.5% Triton X-100) and Hoechst33258 (5 µg/ml). Slices were transferred on superfrost slides, mounted and analyzed using a Leica SP8 confocal microscope.

The following primary antibodies were used: mouse anti-CALBINDIN (1:500; Swant), mouse anti-CALRETININ (1:500; Swant #6B3), rabbit anti-cleaved caspase-3 (1:500; Cell Signalling Technology #9664), rabbit anti-DIO3 (1:25; Pierce #PA5-22886), guinea pig anti-DOUBLECORTIN (1:500; Merck Millipore #AB2253), chicken anti-GFAP (1:500; Covance #PCK591P), rabbit anti-KI67 (1:250; Abcam #ab16667), rabbit anti-LAT1 (1:500; TransGenicInc. #KE026), rabbit-anti LAT2 (1:200; immunoGlobe #0142-10), goat anti-MCM2 (1:100; Abcam #ab4461), rabbit anti-MCT8 (1:500; Sigma-Aldrich #HPA003353), rabbit anti-MCT10 (1:50; MyBioSource #MBS2521988), mouse anti-NEUN (1:300; Millipore #MAB377), rabbit anti-P21 (1:250; Abcam #ab188224), rabbit anti-P27 (1:250; Abcam #ab32034), rabbit anti-P57 (1:200; Sigma #P0357), rat anti-SOX2 (1:500; eBioscience #14-9811-80), rabbit anti-TBR2 (1:250; Abcam #ab183991).

qPCR

To exclude the presence of genomic DNA, one sample without reverse transcriptase was included as well. Ten nanograms of cDNA were employed in one qPCR reaction. Three to four samples per population were subjected to the analysis in duplicate. As a housekeeping gene for normalization Glyceraldehyde 3-phosphate dehydrogenase (Gapdh) was used. The annealing temperature was 55°C for all primer pairs. The following primers were chosen to generate the PCR fragments: *Crym* 5'-GTCCAGGCGTACAGTCACTA-3' and 5'-AGCCTCCTGCACTGATGAAC-3'; *Dio1* 5’-CGTGACTCCTGAAGATGATG-3’ and 5’-CCAATGCCTATGGTTCCTAC-3’; *Dio2* 5’-GATGCTCCCAATTCCAGTGT-3’ and 5’-

AGTGAAAGGTGGTCAGGTGG-3’; *Dio3* 5'-ACTAGGCCACATCGATTC-3' and 5'-CAACCTTTGACTTTCTTTGG-3'; *Dcx* 5'-CTACAGAACCAGAACCTTGC-3' and 5'-TCAGAAGAAACAGCGTACAC-3'; *Gapdh* 5’-ATGCCAGTGAGCTTCCCGTC-3’ and 5’-CATCACCATCTTCCAGGAGC-3’; *Hes5* 5'-GAAACACAGCAAAGCCTTC-3' and 5'-TGGAAGTGGTAAAGCAGC-3'; *Lat1* 5'-GTCGTTCAGTAGCACAGAG-3' and 5'-CCATCTGTCAGTCCAAGTC-3'; *Lat2* 5'-TTCCCATTATCTACCTGCTG-3' and 5'-GAACTCCTGTCAGCATAATG-3'; *Mct8* 5'-TGCCGAATCACAGCCACCAC-3' and 5'-GGCGAATGAGCAGCCACAAC-3'; *Mct10* 5'-GGCCGCATTGCTGACTATTT-3' and 5'-CAATGGGCGCCATGATAGA-3'; *Ncor* 5'-GCAAAGTCGTTATCCTTCAC-3' and 5'-ATGCCTGGCTAACTTCAATA-3'; *Neun* 5'- ATCGGAACCATGTGAAACC-3' and 5'-GTAGCCGGGTGTACGCCAGA-3'; *Oatp1c1* 5'-GCTGTGGAAAACTCAAGGTG-3' and 5'-GGAAGGGATCTCAAACCTTC-3'; *p27kip1* 5'-ACATATCGCTGACTCCATTG-3' and 5'-GCAGATGGTTTAAGAGTGCC-3'; *p57kip2* 5'-CCCCCACTTCATTAGATTGC-3' and 5'-TTATTTAGAACCTGGCGGAC-3'; *Prox1* 5'-TCCGACATCTCACCTTATTC-3' and 5'-TCCGAGAAGTAGGTCTTCAG-3'; *Smrt* 5'-CAACAAGAAACTCAACACCC-3' and 5'-CTTCTACAGGTCATAAGGCC-3'; *Trα1* 5'-GGCAGTTATCTTGTCCCTTT-3' and 5'-CAAGTAAGCACAGACGACTA-3'; *Trα2* 5'-CCGCTTCCTCCACATGAAAG-3' and 5'-CAGCTCTGTCCCTTCTCTCC-3'; *Trβ1* 5'-TGAAGAATGAGCAGACTTCC-3' and 5'-CTTGAGATGCTCTGATCGTT-3'; *Trβ2* 5'-GTAGTTACCCTGGAAACCTG-3' and 5'-GAAAAGGCTTTTCTTCAGGG-3'.

Quantification

Marker positive cells (cleaved caspase-3, CB, CR, DCX, EdU, GFAP, KI67, NEUN, RFP, SOX2, and TBR2) in the SGZ were counted and normalized to the length of the SGZ using ImageJ (NIH). To determine the number of GFAP+/SOX2+ NSCs, broader z-stacks were imaged, and only cells that extended a single GFAP+ process into the granule cell layer were counted. In RFP-labelling experiments, numbers of marker positive/RFP+ cells were counted

and expressed as percentage of all RFP+ cells so as to account for inter-animal variability. In all histo-morphological studies, 5 to 6 images from 3 to 4 sections per animal were quantified.

Relative integrated densities of P21, P27 and P57 immunofluorescence signals were calculated by encircling the nuclei and dividing the value by the analyzed area. Background intensity was measured in the same way by encircling a nucleus-free area in the hilus region, and subtracted from the signals calculated above. 5 pictures per animal and a total of 25 cells (WT vs. MCT8 Ko at P60), 16-25 cells (WT vs. MCT8 KO at P180), and 5-19 cells (control vs. MCT8-NSC KO at P180) per cell type were analyzed.

To evaluate *p27* mRNA levels in the DCX+ population, we reasoned that *Dcx* mRNA levels per cell would be the same in Wt and ko animals even if the NB numbers were different. We therefore calculated the *p27* mRNA/*Dcx* mRNA ratio from dissected hippocampal tissue using RT-PCR protocols described above.

RT-PCR

RNA was isolated, amplified and transcribed into cDNA as above. 12.5 µg cDNA were employed in a standard PCR using an annealing temperature of 55 C and respective qPCR primer pairs. PCR products were visualized on a 3% agarose gel using a G:Box system (Syngene).
