# Supplementary material for: Hippocampal Neurogenesis Requires Cell-Autonomous Thyroid Hormone Signaling
Source: Stem Cell Reports. 2020 Apr 16;14(5):845–60. doi: 10.1016/j.stemcr.2020.03.014 (PMC7220957; doi:10.1016/j.stemcr.2020.03.014)
Supplement: Document S2. Article plus Supplemental Information [file mmc2.pdf]

Stem Cell Reports
Article
ISSCR
OPEN ACCESS

Hippocampal Neurogenesis Requires Cell-Autonomous Thyroid Hormone Signaling

Steffen Mayerl,[1,*] Heike Heuer,[2] and Charles ffrench-Constant[1]
[1]MRC Centre for Regenerative Medicine, University of Edinburgh, 5 Little France Drive, Edinburgh EH16 4UU, UK
[2]University of Duisburg-Essen, University Hospital Essen, Department of Endocrinology, Essen, Germany
*Correspondence: smayerl@exseed.ed.ac.uk or steffen.mayerl@uk-essen.de (S.M.)
https://doi.org/10.1016/j.stemcr.2020.03.014

SUMMARY

Adult hippocampal neurogenesis is strongly dependent on thyroid hormone (TH). Whether TH signaling regulates this process in a cell-autonomous or non-autonomous manner remains unknown. To answer this question, we used global and conditional knockouts of the TH transporter monocarboxylate transporter 8 (MCT8), having first used FACS and immunohistochemistry to demonstrate that MCT8 is the only TH transporter expressed on neuroblasts and adult slice cultures to confirm a necessary role for MCT8 in neurogenesis. Both mice with a global deletion or an adult neural stem cell-specific deletion of MCT8 showed decreased expression of the cell-cycle inhibitor P27KIP1, reduced differentiation of neuroblasts, and impaired generation of new granule cell neurons, with global knockout mice also showing enhanced neuroblast proliferation. Together, our results reveal a cell-autonomous role for TH signaling in adult hippocampal neurogenesis alongside non-cell-autonomous effects on cell proliferation earlier in the lineage.

INTRODUCTION

Adult hippocampal neurogenesis is a highly orchestrated process with cells passing through distinct stages to generate granule cell neurons (GCNs) throughout life (Beckervordersandforth et al., 2015; Kempermann et al., 2004; Remaud et al., 2014). This process is initiated from neural stem cells (NSCs) in the subgranular zone (SGZ) that cycle between quiescence and an activated state in which they generate transiently amplifying precursors (TAPs) from which new post-mitotic neurones are formed via an intermediate neuroblast (NB) state. These newly formed neurons eventually integrate into the existing dentate gyrus granule cell network thereby creating new connections that contribute to CNS plasticity.

A link between hippocampal neurogenesis and cognitive function is well established and adult-onset hypothyroidism is known to result in cognitive perturbations, such as learning and memory deficits (Correia et al., 2009; Miller et al., 2006; Osterweil et al., 1992; Remaud et al., 2014). In light of this, a number of studies have investigated if TH deficiency impairs the hippocampal neurogenic process. These studies have consistently demonstrated an effect on progenitor differentiation and the generation of neurons, but no consistent effects earlier in the lineage on NSC behavior (Ambrogini et al., 2005; Desouza et al., 2005; Montero-Pedrazuela et al., 2006). However, a key question that remains unanswered is whether this effect results from a cell-autonomous requirement for TH signaling within the hippocampal lineage or from an indirect, non-cell-autonomous effect resulting from TH function in supporting glial and other cell types. Addressing this is important to identify the necessary cellular targets for therapies designed to treat age-related cognitive decline based on modulated TH signaling.

One strategy to address this question is to identify essential components of the TH signaling pathway selectively expressed in NBs and then compare the effects of global and conditional knockouts of these components. The latter will reveal only cell-autonomous effects, while the former will reveal both cell- and non-cell-autonomous effects. By examining the cellular expression pattern of components of the TH signaling pathway throughout the adult hippocampal neurogenic program we identified such a component, the TH transporter monocarboxylate transporter 8 (MCT8). Transgenic mice lacking MCT8 either globally or just in the hippocampal neurogenic lineage both showed impaired differentiation of NBs and a reduced formation of new GCNs in the adult hippocampus. This impairment is associated with an improper regulation of the cell-cycle inhibitor P27KIP1 in neural progenitors. We conclude that the effect of TH on the generation of neurons from NBs is cell-autonomous and that MCT8 is a critical gatekeeper for this step of hippocampal neurogenesis.

RESULTS

Differential Expression of TH Signaling Components within the Hippocampal Neurogenic Lineage

TH signaling in the CNS is regulated at several levels. First, TH transporters, such as the L-type amino acid transporters (LAT) 1 and 2, organic anion transporting polypeptide (OATP) 1C1, and MCT8 and MCT10, are mandatory for TH transmembrane passage across the blood-brain barrier (BBB) and cellular TH uptake (Bernal et al., 2015; Heuer

Check for updates

This is an open access article under the CC BY license (http://creativecommons.org/licenses/by/4.0/).

and Visser, 2013). Second, intracellular iodothyronine deiodinases (DIO) then either activate (DIO2) or inactivate (DIO3) TH (Bianco et al., 2002). Third, μ-Cristallin (CRYM), a cytosolic TH binding protein, can regulate intracellular TH levels (Suzuki et al., 2007). Fourth, nuclear TH receptors (TRs) encompassing the ligand binding isoforms TRα1, TRβ1, and TRβ2, as well as non-ligand binding isoforms such as TRα2, regulate gene expression in response to TH (Flamant and Gauthier, 2013; Koenig et al., 1989). Last, co-activators or co-repressors are recruited to TR isoforms, including NCOR (nuclear receptor corepressor; NCOR1) and SMRT (silencing mediator of retinoid and thyroid hormone receptors; NCOR2) (Astapova and Hollenberg, 2013).

To unravel the temporal expression pattern of these TH signaling components in the adult mouse hippocampus and identify any selectively expressed in NBs, we microdissected and dissociated dentate gyri for fluorescence-activated cell sorting (FACS). We used intracellular markers to isolate different progenitor/neuronal populations that develop in sequence within the hippocampal neurogenic lineage (Kempermann et al., 2004) (Figure 1A). The first cell population comprises NSCs that are located in the SGZ of the dentate gyrus, extend a radial process into the molecular layer, and are positive for glia fibrillary acidic protein (GFAP), SRY-Box 2 (SOX2), and NESTIN. The second population encompasses TAPs (intermediate progenitors; type 2a and type 2b progenitors), which express the transcription factor T-box brain protein 2 (TBR2) and are generated by asymmetrical division of activated NSCs. This population can be subdivided by expression of the neuronal differentiation-promoting factor PROX1 (prospero homeobox 1) and the immature neuronal marker doublecortin (DCX) in type 2b progenitors. Cells of the third population, DCX+ type 3 NBs, develop a vertical process while exiting the cell cycle to generate, fourth, immature post-mitotic neurons (INs), which are characterized by transient expression of the calcium-binding protein calretinin (CR). Finally, the fifth population comprises GCNs in which DCX and CR expression cease and calbindin (CB) expression is initiated.

Using forward and side scatter, we separated cells (P1; 2.1%–8.0%) from debris and selected single cells (P2; 94.9%–98.9%) (Figure S1A). Single cells viable before fixation were identified based on a low intensity of a fixable live/dead cell stain (P3; 38.4%–53.4%). From those cells, a TBR2+ population was isolated (0.6%–2.3%) (Figure S1B). The TBR2− population (P4) was then subdivided into a DCX− and a DCX+ population (4.1%–7.8%). The latter was then sorted into CR− NBs (51.1%–92.4%) and into CR+ INs (5.9%–42.6%). In a second sorting strategy, CB+ GCNs (5.5%–21.3%) were isolated from live cells (P3) (Figure S1C). From the CB− population (P4) NESTIN+/GFAP+ NSCs were sorted (1.1%–5.2%). All other cells were collected for RIN (RNA integrity number) value determination. To preserve RNA integrity, we performed staining and sorting steps at low temperatures and in the presence of RNase inhibitor. As shown in Figure S1D comparing the RIN value of a fixed sample, a fixed/stained sample and cells undergoing the staining/sorting procedure, a RIN value of 7.0 or higher was reached with our measures.

We then performed qPCR on isolated populations after mRNA amplification. To validate the identity of the isolated cell populations, neurogenic marker expression was analyzed (Figure 1B). The stem cell marker *Hes5* (Beckervordersandforth et al., 2015) was strongly expressed in NSCs. As expected, we found high *Dcx* mRNA expression in TAPs, NBs, and INs. *Prox1* transcript was expressed in NB, IN, and GCN samples. *NeuN* mRNA, although detectable in TAP and NB, was highly enriched in GCN samples. NSC, NB, and GCN samples were also used for RT-PCR (Figure S1E). *Dcx* was again enriched in the NB population, while the lineage marker *Prox1* was found in both NBs and GCNs.

Next, we assessed the mRNA expression profile of TH signaling components. Within the TH transporters (Figure 1C), we observed *Mct8* transcripts primarily in NBs and GCNs, while *Mct10* mRNA was enriched in mature neurons. *Lat1* and *Lat2* expression was detected in NSCs and TAPs, whereas only *Lat2* was further enriched in GCNs. Analysis of TR expression profiles revealed *Trα1*, *Trα2*, *Trβ1*, and *Trβ2* transcripts in the hippocampal lineage (Figure 1D). While both *Trα* isoforms and *Trβ1* mRNAs were predominantly expressed in NSC, NB, and GCN populations, *Trβ2* transcript levels were downregulated upon neuronal maturation. Finally, *Crym*, *Dio3*, *Ncor*, and *Smrt* exhibited a similar profile of transcripts with peaks in NB and GCN stages (Figure 1E), matching the expression of *Mct8*, *Trα1*, and *Trα2*. Of note, *Oatp1c1*, *Dio1*, and *Dio2* transcripts were not detected in the analyzed cell populations.

To complement our qPCR analysis, we performed immunofluorescence studies using perfusion-fixed brain cryosections from 2-month-old animals and commercially available antibodies against DIO3, LAT1, LAT2, MCT8, and MCT10 in combination with cell-type-specific markers (Figure 2). In contrast to our qPCR results, LAT1 co-localized only with the endothelial cell marker CD31/PECAM-1 throughout the dentate gyrus (Figure S2), while none of the proteins above could be detected in GFAP+/SOX2+ NSCs (Figure 2A). No co-localization with the proliferation marker MCM2 present in activated NSCs, TAPs, and cycling NBs was observed for any component except MCT8, which was found in a specific subset of MCM2+ cells also expressing DCX (Figure 2B). By using a triple-staining protocol, we observed strong expression of MCT8 protein in DCX+/CR−

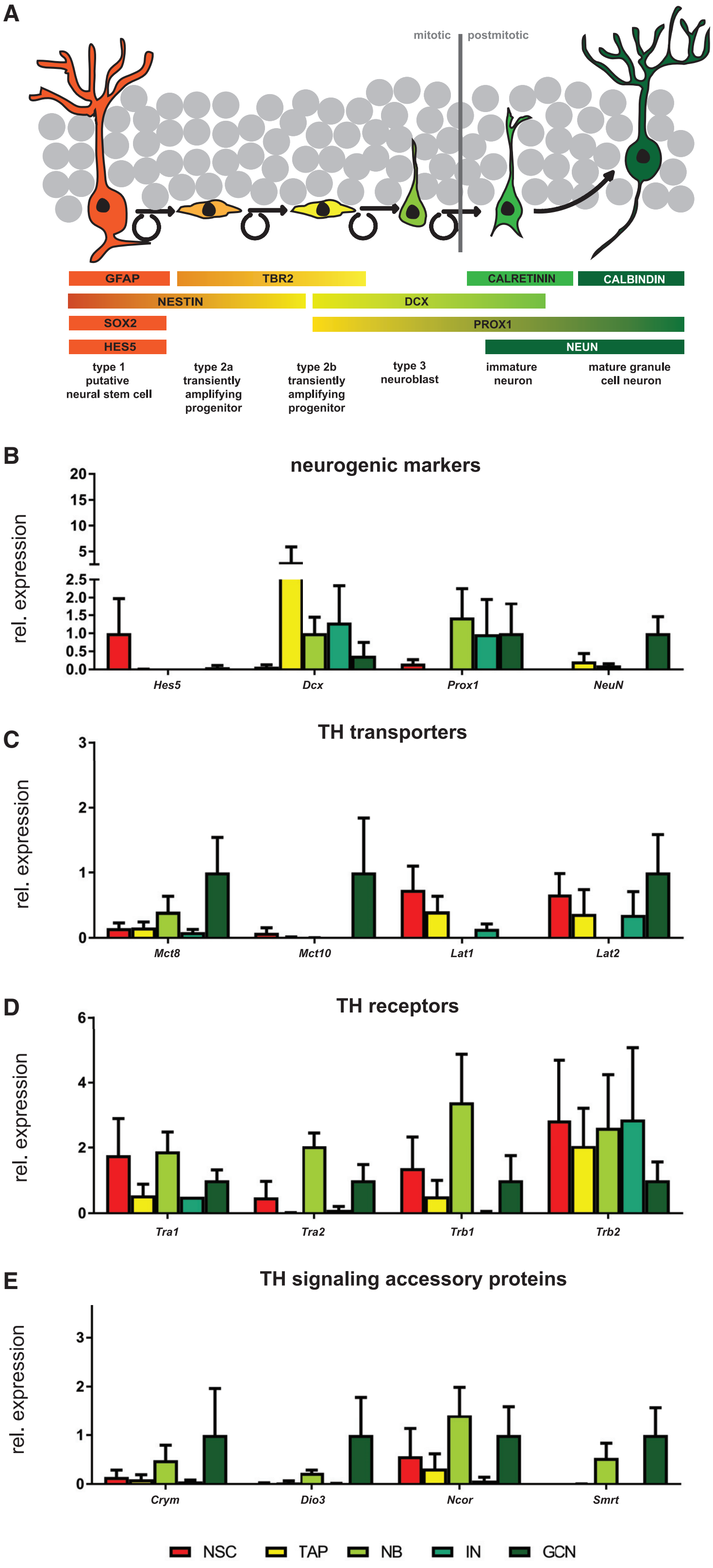

Figure 1. Alterations in mRNA Expression of TH Signaling Components during Adult Hippocampal Neurogenesis

Micro-dissected dentate gyri were subjected to FACS and neurogenic/neuronal populations were sorted according to their expression of intracellular markers.

(A) Schematic representation of the hippocampal neurogenic program illustrating expression of stage-specific markers used for sorting and validation strategies.

(B–E) (B) qPCR analysis of neurogenic markers showing that isolated populations are of the expected identity. Relative mRNA expression of (C) TH transporters, (D) TH receptor isoforms, and (E) accessory proteins are depicted. Transcript levels were normalized to *Gapdh* expression and transcript expression in GCN. Note that, due to their absence from GCN samples, *Hes5* and *Lat1* values were normalized to NSC levels while *Dcx* expression was normalized to NB values. n = 2–4 individual samples per cell population. Group means + SEM are shown.

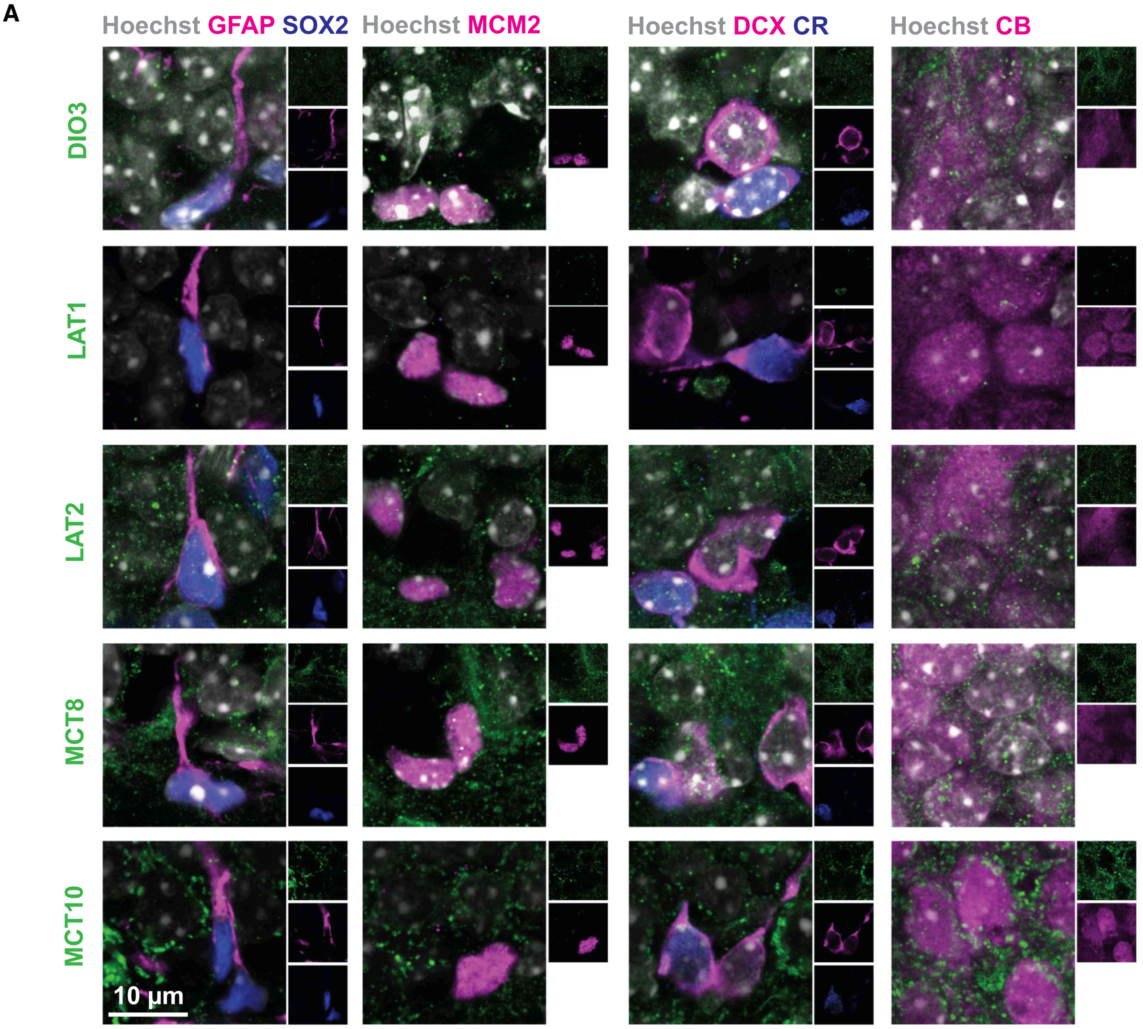

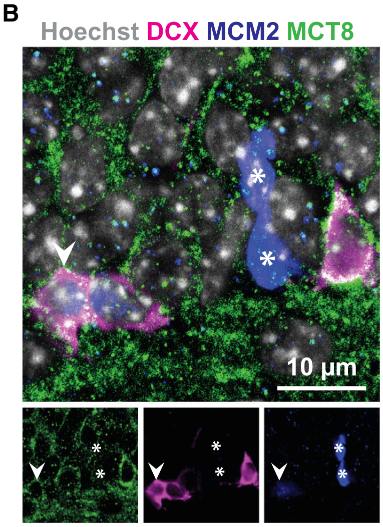

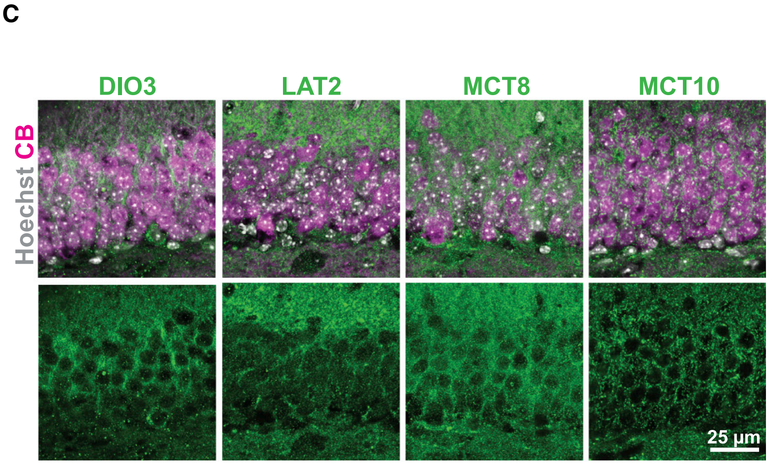

(legend on next page)

NBs and in DCX+/CR+ INs (Figure 2A) while none of the other proteins showed detectable expression at this stage. In agreement with our qPCR results, CB+ GCNs were positive for DIO3, LAT2, MCT8, and MCT10 protein. Whereas MCT8 and MCT10 exhibited equal expression throughout the granule cell layer, an asymmetrical pattern was found for DIO3 and LAT2 with stronger signals in the region contacting the molecular layer of the hippocampus (Figure 2C). We conclude that MCT8 is present in NBs, while later stages of the lineage contain a wider range of transporters. As TH transporters are essential for TH signaling, this finding identifies MCT8 as a possible target for our global and conditional knockout strategy to define the cell autonomy of TH signaling during the generation of neurons from NBs.

Inhibition of MCT8 Reduces the Formation of New Neurons in Adult Hippocampal Slices

Before generating transgenic mice, we sought to confirm a functional role for MCT8 in hippocampal neurogenesis using commercially available inhibitors *in vitro*. For that purpose, we established a protocol to maintain adult hippocampal slices for at least 3 weeks by combining different published protocols (Kim et al., 2013; Kleine Borgmann et al., 2013) and adding indomethacin for its protective effects on neurogenesis *in vivo* and *ex vivo* (Gerlach et al., 2016; Melo-Salas et al., 2018). As a readout, we performed EdU-tracing studies together with KI67 labeling to quantify progenitor proliferation, DCX labeling to quantify type 2b progenitors, NBs, and INs, and NEUN staining for neurons (Figure 3A).

Incubation with the MCT8-specific inhibitor silychristin (Johannes et al., 2016) for 24 h did not alter proliferation in the SGZ (Figure S3A) or the number of EdU+ and KI67+/EdU+ cells (Figures 3B and 3C). By 7 days in culture a trend toward decreased formation (EdU+, Figure 3D) and proliferation (KI67+/EdU+, Figure S3A) of DCX+ cells in silychristin-treated slices was seen, while longer incubation with the inhibitor resulted in a significant reduction of newly formed neurons (NEUN+/EdU+) after 21 days (Figure 3E). By contrast, treatment of hippocampal slices with the LAT inhibitor BCH (Ritchie et al., 1999, 2003) or the deiodinase inhibitor iopanoic acid (Dentice et al., 2013) had no effect on proliferation or NB and neuron formation (Figures S3B and S3C). These inhibitor studies point to a crucial role of MCT8 during later stages of hippocampal neurogenesis.

Absence of MCT8 *In Vivo* Compromises Adult Hippocampal Neurogenesis

Having confirmed a functional role for MCT8 *in vitro*, we assessed the consequences of global inactivation of MCT8 *in vivo*. At the age of 2 months, overall NSC numbers (defined as GFAP+/SOX2+ cells extending a radial process into the granule cell layer) were the same in MCT8 knockout (KO) and wild-type (WT) littermates. However, NSC activation was impaired in MCT8 KO mice as significantly fewer NSCs were labeled by the proliferation marker KI67 (Figure 4A). Total numbers of KI67+ progenitors and the density of KI67+/DCX+ type 2b and type 3 progenitors were similar between the genotypes (Figure 4B). Likewise, no difference in the number of TBR2+ TAPs (Figure S4A), cleaved caspase-3+ apoptotic cells (Figure S4B) and DCX+/CR− NBs (Figure 4C) could be observed. In contrast, a significantly reduced number of DCX+/CR+ INs was found in the MCT8 KO SGZ (Figure 4C) pointing to impaired differentiation.

To explore the dynamics of neurogenesis further, we performed EdU label-retention studies. We injected 2-month-old mice with EdU and followed the formation of progenitor cells and neurons by NSCs in the hippocampus (Figure 4D) either at day 3 post injection (3 dpi) to examine the level of proliferation within each population or 28 dpi to quantify the number of labeled cells that are fully differentiated. At 3 dpi, we detected significantly more EdU-labeled KI67+/DCX+ progenitors and DCX+/CR− NBs in MCT8 KO mice compared with WT littermates (Figures 4E and 4F, respectively), whereas numbers of DCX+/CR+/EdU+ newly formed INs were not different (Figure 4F). At 28 dpi, significantly fewer EdU+/CB+ GCNs were seen in MCT8 KO mice (Figure 4G), demonstrating that dividing NBs exhibit differentiation impairments in the absence of MCT8.

Hippocampal neurogenesis is highly active in young animals and rapidly declines with age, being already greatly compromised at around 6 months of age (Ben Abdallah et al., 2010; Kuhn et al., 2018). We wondered if the deficits

Figure 2. Spatiotemporal Protein Expression of TH Signaling Components

(A) Perfusion-fixed coronal forebrain cryosections were immunostained to visualize DIO3, LAT1, LAT2, MCT8, and MCT10 protein (green) in the SGZ in GFAP+ (magenta)/SOX2+ (blue) NSCs, in MCM2+ (magenta) proliferating cells, in DCX+ (magenta) cells either negative for CR (type 2b progenitors and NBs) or CR+ (blue; INs), and in CB+ (magenta) GCNs. Nuclei were stained with Hoechst 33258 (gray).

(B) MCT8 (green) co-stained with MCM2 (blue) in DCX+ (magenta; arrowhead) but not DCX− (*) cells. Cell nuclei counterstained by Hoechst 33258 are displayed in gray.

(C) Distribution of DIO3, LAT2, MCT8, and MCT10 (green) over the height of the suprapyramidal blade of the dentate gyrus. Nuclei were stained for Hoechst 33258 (gray) and mature GCNs were identified by CB (magenta). DIO3 and LAT2 protein are asymmetrically distributed with higher signal intensities in areas close to the molecular layer, while MCT8 and MCT0 appear evenly dispersed.

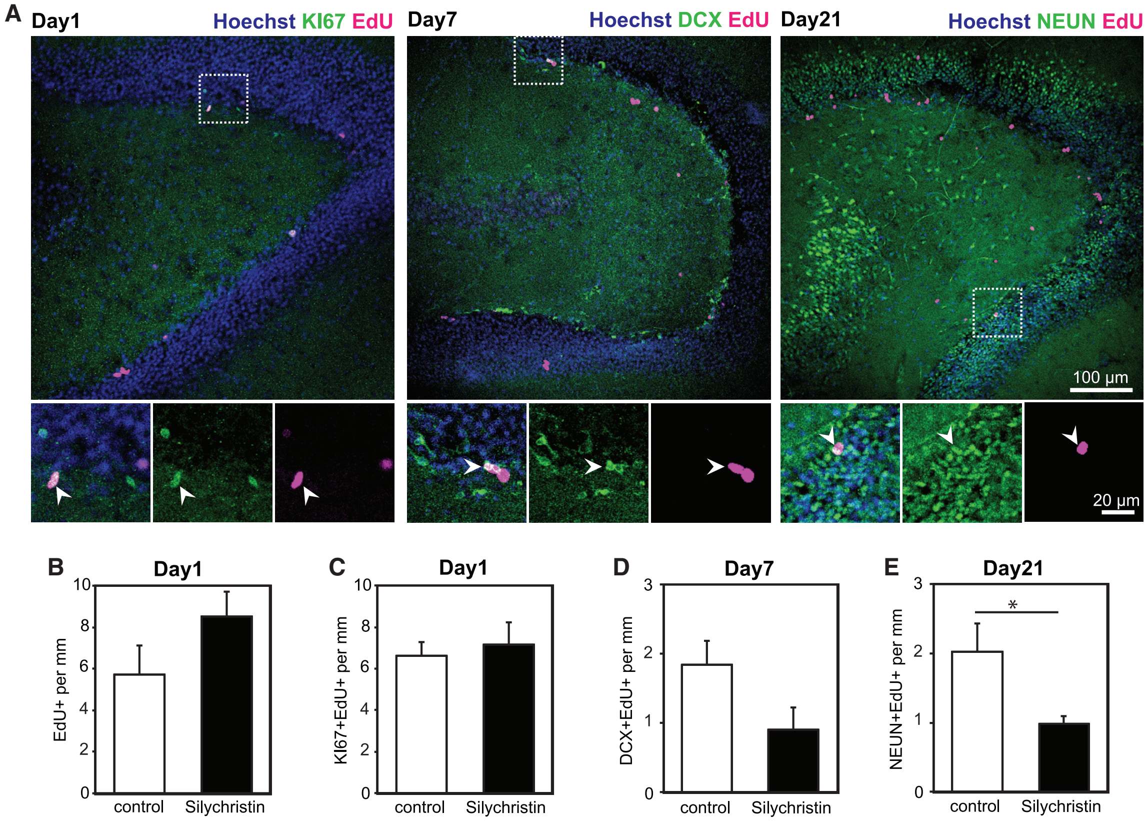

Figure 3. Inhibition of MCT8 in Adult Slices Perturbs Neuron Formation

After acute EdU injection, adult brain slices cultured *ex vivo* for up to 3 weeks. Lineage progression in the presence of TH signaling inhibitors was assessed.

(A) Exemplar pictures from adult slices grown in control medium. EdU incorporation (magenta) into proliferating cells (KI67+, green) was visualized after 1 day; into DCX+ cells (type 2b progenitors, NBs and INs) after 7 days; and into newly formed neurons (NEUN+, green) after 21 days. Hoechst 33258-counterstained nuclei are shown in blue. Total number of EdU+ cells (B) and incorporation of EdU into KI67+ cells (C) after 1 day in culture, EdU+/DCX+ cells at day 7 (D) and newly formed neurons (EdU+/NEUN+) at day 21 (E) upon exposure to 25 µM silychristin were quantified. n = 4–6 mice per condition. Group means + SEM are shown.*, $p < 0.05$, unpaired two-tailed Student's t test.

resulting from loss of MCT8 are also observed at older ages when the number of cells transitioning from NB to neuron populations is reduced. To address this, we repeated our analysis at 6 months using the same EdU injection paradigm as in Figure 4D. At this age, MCT8 KO mice exhibited a slight but significantly increased density of GFAP+/SOX2+ cells with a radial process in the SGZ, while similar numbers of NSCs were labeled with KI67 (Figure 5A). No differences were observed in the number of TBR2+ TAPs (Figure S5A), cleaved caspase-3+ apoptotic cells (Figure S5B), or the total number of KI67+ cells. Although the increase in KI67+/DCX+ cells did not reach statistical significance (Figure 5B), the number of DCX+/CR− cells in the SGZ (comprising NBs and type 2b cells) was almost doubled in MCT8 KO mice (Figure 5C). In contrast, MCT8 KO mice demonstrated a severely reduced formation of new GCNs (CB+/EdU+) when assessed at 28 dpi (Figure 5D). We conclude that the deficit in neuron differentiation is also present in older animals despite the overall reduction in neurogenesis.

As a second approach to follow the progeny of dividing NSCs, we used a stable labeling strategy by generating WT and MCT8 KO mice expressing a *Nestin-CreERT2* construct and a *tdTomato* reporter (hereafter *Rfp*) (Figure 5E). Following 5 consecutive days of tamoxifen treatment at the age of 4 weeks, mice were kept for 5 months before analysis, so matching the 6-month time point used in the EdU analysis above. A similar absolute number of NSCs was labeled in both genotypes, although their relative contribution to all RFP+ cells was significantly higher in MCT8 KO mice (Figure 5F). Despite this, and in agreement with our EdU incorporation studies, the relative number of NSC-derived RFP+/CB+ cells among all RFP+ cells was significantly reduced in MCT8 KO mice. Together,

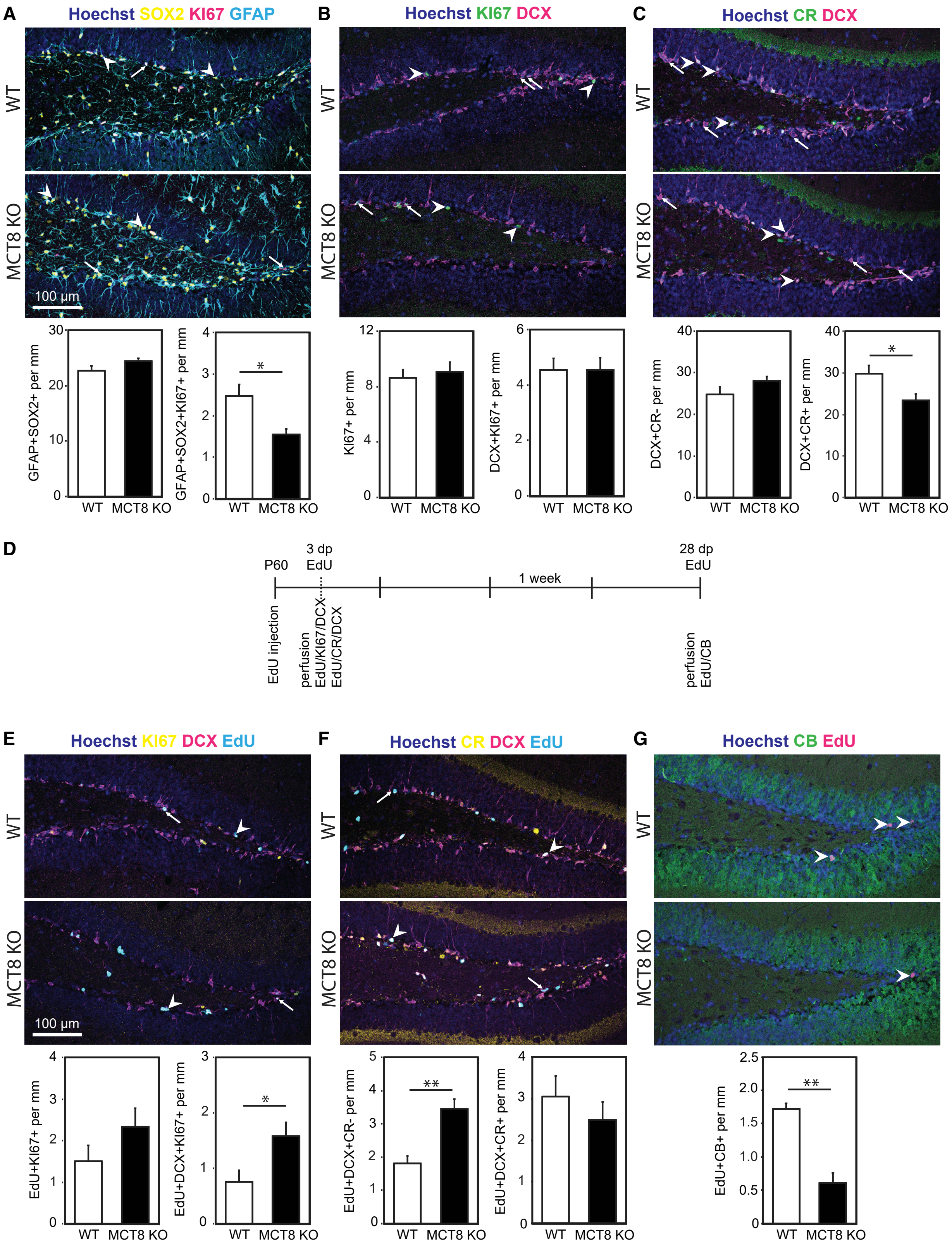

(legend on next page)

our experiments using different labeling techniques and ages confirm that the absence of MCT8 and thus the loss of TH transporter activity in NBs inhibits the generation of new GCNs in the dentate gyrus.

The Deficit in Neuron Formation Caused by MCT8 Loss Is Cell-Autonomous

These deficits in neurogenesis may result from cell-autonomous effects of MCT8 deletion in hippocampal NBs. Alternatively, the well-described endocrine alterations after global loss of MCT8, such as high serum T3 and low serum T4 levels and/or impaired transport of T3 across the BBB, which in turn causes a mild central TH deficiency (Ceballos et al., 2009; Trajkovic et al., 2007), may impact NB differentiation and GCN formation. To distinguish between these possibilities, we generated a mouse model with specific deletion of MCT8 in the adult neurogenic lineage (Figure 6A). To this end, *Mct8*+/fl females were mated with males heterozygous for the *Nestin-CreERT2* allele and homozygous for a *tdTomato* reporter allele (hereafter *Rfp*) (Figure 6A). *Mct8+/y*, *Nestin-CreERT2/+*, *Rfp/+* (control), and *Mct8fl/y*, *Nestin-CreERT2/+*, *Rfp/+* (MCT8-NSC KO) mice were used (note that the *Mct8* gene is located on the X chromosome). Tamoxifen-induced Cre-activation at 1 month of age resulted in RFP expression and deletion of MCT8 in MCT8-NSC KO animals in adult NSCs and thus the neurogenic lineages only as confirmed by the loss of MCT8 expression in RFP+ neurons in MCT8-NSC KO mice (Figure S6A). All analyses were performed 5 months later to match the 6-month time point of global MCT8 KO mice which showed GCN formation impairments and higher NB numbers. Again, we corrected our analysis for differences between individual animals by normalizing cell counts to the overall number of RFP+ cells. We found no differences in the percentage of RFP+ NSCs (Figure 6B), activated NSCs (GFAP+/RFP+/KI67+/radial process; Figure 6C), proliferating cells (KI67+/RFP+), proliferating type 2b cells/NBs (DCX+/KI67+/RFP+) (Figure 6D), TBR2+/RFP+ TAPs (Figure S6B), and apoptotic cells (caspase-3+/RFP+; Figure 6E). In contrast to age-matched global MCT8 KO mice in which NB numbers were increased, we detected a similar number of NBs (DCX+/CR−/RFP+) in MCT8-NSC KO and controls at 6 months of age alongside a trend toward fewer INs (DCX+/CR+/RFP+) (Figure 6F). As in global MCT8 KO mice, however, the relative number of CB+/RFP+ GCNs was significantly decreased in MCT8-NSC KO (Figure 6G), confirming a cell-autonomous role of MCT8 within the hippocampal neurogenic lineage.

Expression of the Cell-Cycle Inhibitor P27KIP1 Is Impaired in MCT8 Deficiency

One mechanism by which TH induces differentiation is by suppression of the cell cycle (Remaud et al., 2014), with direct regulation of cell-cycle inhibitors, such as cyclin-dependent kinase inhibitor 1B (CDKN1B; P27KIP1) (García-Silva et al., 2002; Holsberger et al., 2003). To examine this mechanism in the hippocampal neurogenic lineage, we quantified P27KIP1 levels in DCX+/CR− NBs and DCX+/CR+ INs in 2-month-old WT and MCT8 KO mice (Figure 7A). We discovered significantly reduced P27 fluorescence intensities in both cell populations in MCT8 KO animals and replicated this finding in both 6-month-old MCT8 KO mice (Figure 7B) and 6-month-old MCT8-NSC KO mice (Figure 7C). As the CIP/KIP family of cell-cycle/CDK inhibitors comprises two more members, P21CIP1/WAF1 (CDKN1A) and P57KIP2 (CDKN1C), we assessed their expression in 2- and 6-month-old WT and MCT8 KO mice, but failed to observe differences in P21 (Figures 7D and 7E, respectively) and P57 (Figures 7F and 7G, respectively) immunofluorescence levels. Likewise, *p27kip1/Dcx* transcript ratios were reduced in micro-dissected dentate gyri from 6-month-old MCT8 KO mice, while *p57* mRNA levels were not different (Figure S7). In summary, our results show a specific decrease in P27KIP1

Figure 4. Adult Hippocampal Neurogenesis Is Altered in 2-Month-Old MCT8 KO Mice
Perfusion-fixed cryosections of 2-month-old WT and MCT8 KO littermates were immunostained for stage-specific markers of hippocampal neurogenesis.
(A) Total numbers of GFAP+ (cyan) and SOX2+ (yellow) NSCs (arrowheads) harboring a single process protruding into the granule cell layer, and activated NSCs (KI67+; magenta; arrows).
(B) Overall numbers of proliferating cells in the SGZ (all KI67+ cells; green; arrowheads) and numbers of proliferating type 2b/NBs expressing KI67 and DCX (magenta; arrows).
(C) DCX (magenta) and CR (green) were used to discriminate between type 2b progenitors/NBs (only DCX+; arrows) and INs (DCX+/CR+; arrowheads).
(D) Timeline of EdU-labeling experiments. Injected at P60, mice were perfused 3 or 28 days later and stained as shown.
(E) EdU (cyan) retention in proliferating progenitors (KI67+; yellow; arrowheads) and DCX+ (magenta; arrows) cells was assessed at 3 dpi.
(F) Incorporation of EdU (cyan) into type 2b progenitors/NBs (DCX+ [magenta]/CR− [yellow]; arrows) and into INs (DCX+/CR+; arrowheads).
(G) 28 days after EdU pulse, GCN formation (CB+ [green] and EdU [magenta]) was analyzed. In all experiments, cell nuclei were counterstained with Hoechst 33258 (blue). n = 4 (28 dp EdU) or n = 6 (3 dp EdU) mice per genotype. Group means + SEM are shown.*, $p < 0.05$; **, $p < 0.01$, unpaired two-tailed Student's t test.

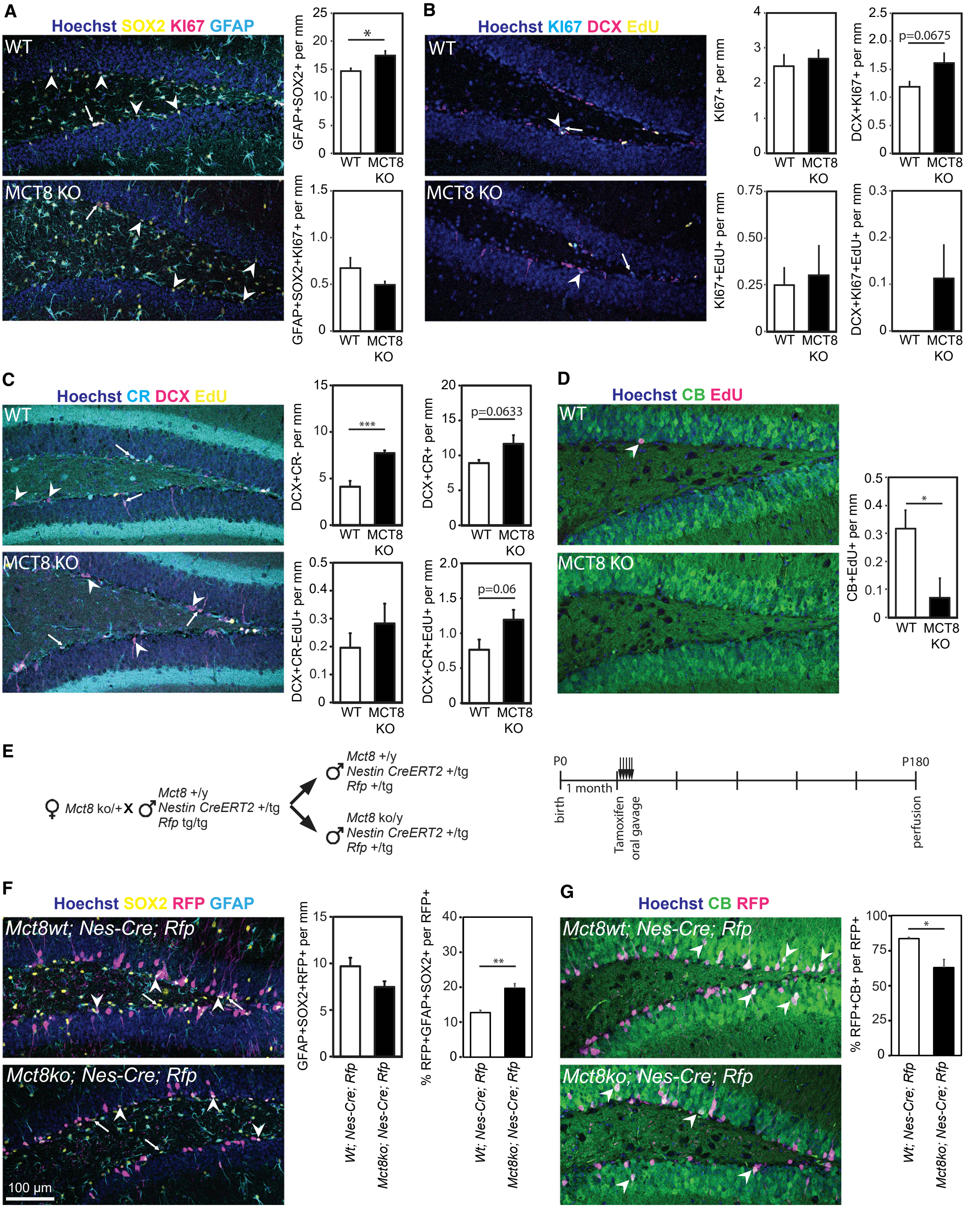

(legend on next page)

after loss of MCT8 in the hippocampal neurogenic lineage, which likely underlies the impaired differentiation capacities in MCT8 deficiency.

DISCUSSION

Patients with adult-onset hypothyroidism show specific defects in hippocampal memory function and a decreased hippocampal volume (Cooke et al., 2014; Correia et al., 2009; Ittermann et al., 2018). These clinical findings may be explained by a profound impact of TH on hippocampal neurogenesis, a process imperative for learning and memory, as animal experiments have confirmed that TH deficiency delays neuronal differentiation and perturbs the birth of new GCN in the adult hippocampus (Ambrogini et al., 2005; Desouza et al., 2005; Montero-Pedrazuela et al., 2006). However, the widespread systemic effects of TH deficiency make it impossible from these experiments using globally hypothyroid animals to resolve the key question if TH signaling impairs NB differentiation cell-autonomously. Here, we demonstrate such a cell-autonomous effect by using a conditional knockout strategy following a comprehensive analysis of the cell-specific repertoire of TH transporters, receptors, and metabolizing enzymes during hippocampal neurogenesis. A combination of FACS and qPCR allowed us to analyze distinct cell populations within the neurogenic program. With this approach, we could confirm the presence of *Trα*, *Trβ1*, and *Trβ2* transcripts in cycling progenitors and GCNs as well as *Trα1* expression in DCX+ progenitors as described before (Desouza et al., 2005; Kapoor et al., 2010). We also demonstrated the presence of DIO3 and the TH transporters LAT2, MCT8, and MCT10 in GCNs. Critically, however, of all TH transporters analyzed only MCT8 was found to be expressed in NBs at both the mRNA and protein level, suggesting a distinct function within the TH-regulated neurogenic program and enabling its manipulation as a means of addressing the central question of this study.

To our knowledge, this is the first study using a conditional knockout strategy to investigate TH signaling in the hippocampal lineage. The importance of this approach is highlighted by a comparison with a global MCT8 KO mouse model. The critical role of MCT8 in transporting T3 and T4 across brain barriers (Bernal et al., 2015; Ceballos et al., 2009; Trajkovic et al., 2007) means that the brain of global MCT8 KO mice is in a mild hypothyroid state, affecting TH metabolism and regulation of TH target genes. That this causes non-cell-autonomous effects on neurogenesis is shown by our finding that at 6 months of age, the number of NBs was not altered in MCT8-NSC KO mice while it was increased in global MCT8 KO mice. This increase cannot simply be explained by a hypothyroid neurogenic niche due to a global loss of MCT8. Both TRα1 mutant mice that show features of a hypothyroid CNS and globally hypothyroid animals exhibit a reduced number of DCX+ cells in the SGZ (Kapoor et al., 2010; Montero-Pedrazuela et al., 2006), while an increase in NB numbers was reported in TRα1 KO, hyperthyroid TRβKO, or T3-treated mice (Kapoor et al., 2010, 2011, 2012). In the latter model, increased BrdU labeling of DCX+ cells was attributed to earlier acquisition of DCX immunoreactivity (Kapoor et al., 2012). The increased EdU labeling in DCX+ cells we observe in MCT8 KO animals may be explained in the same way, linked to the hyperthyroid periphery of MCT8 KO mice (Trajkovic et al., 2007). In keeping with this, we do not see earlier DCX expression in slices treated with an MCT8 inhibitor, where the level of TH in the culture medium is normal. However, alterations in cell-cycle entry or kinetics cannot be fully excluded.

Non-cell-autonomous effects earlier in the lineage may also explain the differences observed in NSC and NB numbers at 2 and 6 months between MCT8 KO and WT animals. Reduced NSC activation in the global KO at 2 months

Figure 5. MCT8 Deficiency Compromises Adult Hippocampal Neurogenesis at 6 Months of Age
Neurogenesis was assessed in 6-month-old males.
(A) Numbers of GFAP+ (cyan)/SOX2+ (yellow) NSCs with a radial process (arrowheads) as well as density of activated KI67+ (magenta; arrow) NSCs.
(B) Proliferation 3 days after EdU injection. Overall KI67+ (cyan; arrows) and KI67+/EdU+ (yellow) cell numbers are shown. Late-stage proliferating cells expressing DCX (magenta; arrowheads) show a higher proliferative capacity.
(C) Density of DCX+ (magenta)/CR− (cyan) type 2b progenitors/NBs (arrowheads) as well as of DCX+/CR+ INs (arrows) with or without EdU (3 dpi; yellow).
(D) Newly formed GCNs (arrowheads) positive for CB (green) and EdU (magenta) were visualized 28 days after EdU injection.
(E) Breeding strategy to generate males harboring the WT or *Mct8* KO allele as well as *Nestin-CreERT2* and *Rfp* reporter transgenes. Animals were gavaged for 5 consecutive days at 4 weeks of age and perfused at 6 months of age.
(F) Numbers of GFAP+ (cyan)/SOX2+ (yellow) NSCs (arrows) with a radial process and of RFP+ (in magenta; arrowheads) NSCs were counted and quantified as per mm and percentage of RFP+ cells.
(G) RFP+ (magenta)/CB+ (green) GCNs (arrowheads) were counted and normalized to the number of RFP+ cells. Cell nuclei were stained with Hoechst 33258 (blue). n = 4 (28 dp EdU and *Nestin-Cre; Rfp* animals) or n = 6 (3 dp EdU injection) mice per genotype. Group means + SEM are shown.*, $p < 0.05$; **, $p < 0.01$; ***, $p < 0.001$, unpaired two-tailed Student's t test.

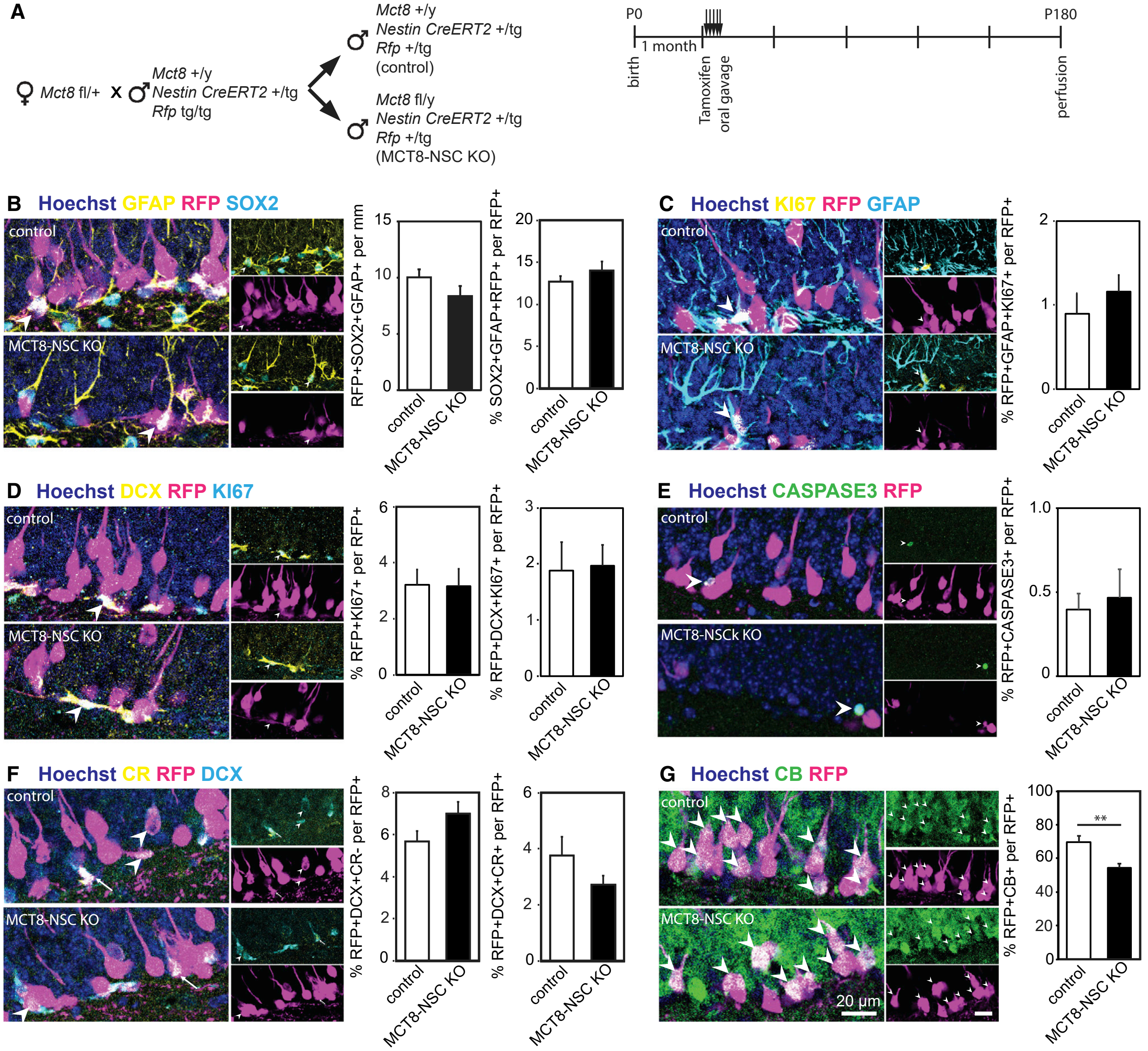

Figure 6. Absence of MCT8 in NSCs Compromises Adult Hippocampal Neurogenesis

(A) *Mct8*fl/+ females were bred with males carrying *Nestin-CreERT2* and *Rfp* reporter alleles to generate *Mct8*+/y, *Nestin-CreERT2*, *Rfp* (control), and *Mct8fl*/y, *Nestin-CreERT2*, *Rfp* (MCT8-NSC KO) littermates. Tamoxifen was given for 5 consecutive days at 4 weeks of age and animals were perfused at 6 months of age.

(B) Number of RFP+ (magenta)/GFAP+ (yellow)/SOX2+ (cyan) NSCs per mm SGZ and their percentage contribution to all RFP+ cells was determined.

(C–F) Relative numbers of RFP+ (magenta; arrowheads)-labeled activated NSCs (KI67+ [yellow]/GFAP+ [cyan]) (C), proliferating cells (KI67+; cyan) and proliferating DCX+ (yellow) cells (D), apoptotic cells (caspase-3+; green) (E), DCX+ (cyan)/CR−(yellow) type 2b progenitors/NBs, and RFP+/DCX+/CR+ INs (arrows) (F).

(G) Ratio of RFP+ (magenta)/CB+ (green) GCNs (arrowheads) over all RFP+ cells. Hoechst 33258-labeled cell nuclei are depicted in blue. n = 5 mice per genotype. Group means + SEM are shown.**, $p < 0.01$, unpaired two-tailed Student's t test.

(which as it is not seen in the MCT8-NSC KO mice must be a non-cell-autonomous effect of TH signaling) would be expected to preserve the NSC population and so explain the increased NSCs present in the MCT8 KO at 6 months as compared with WT mice at the same age. This in turn could attenuate the normal age-related decline in NBs (as would

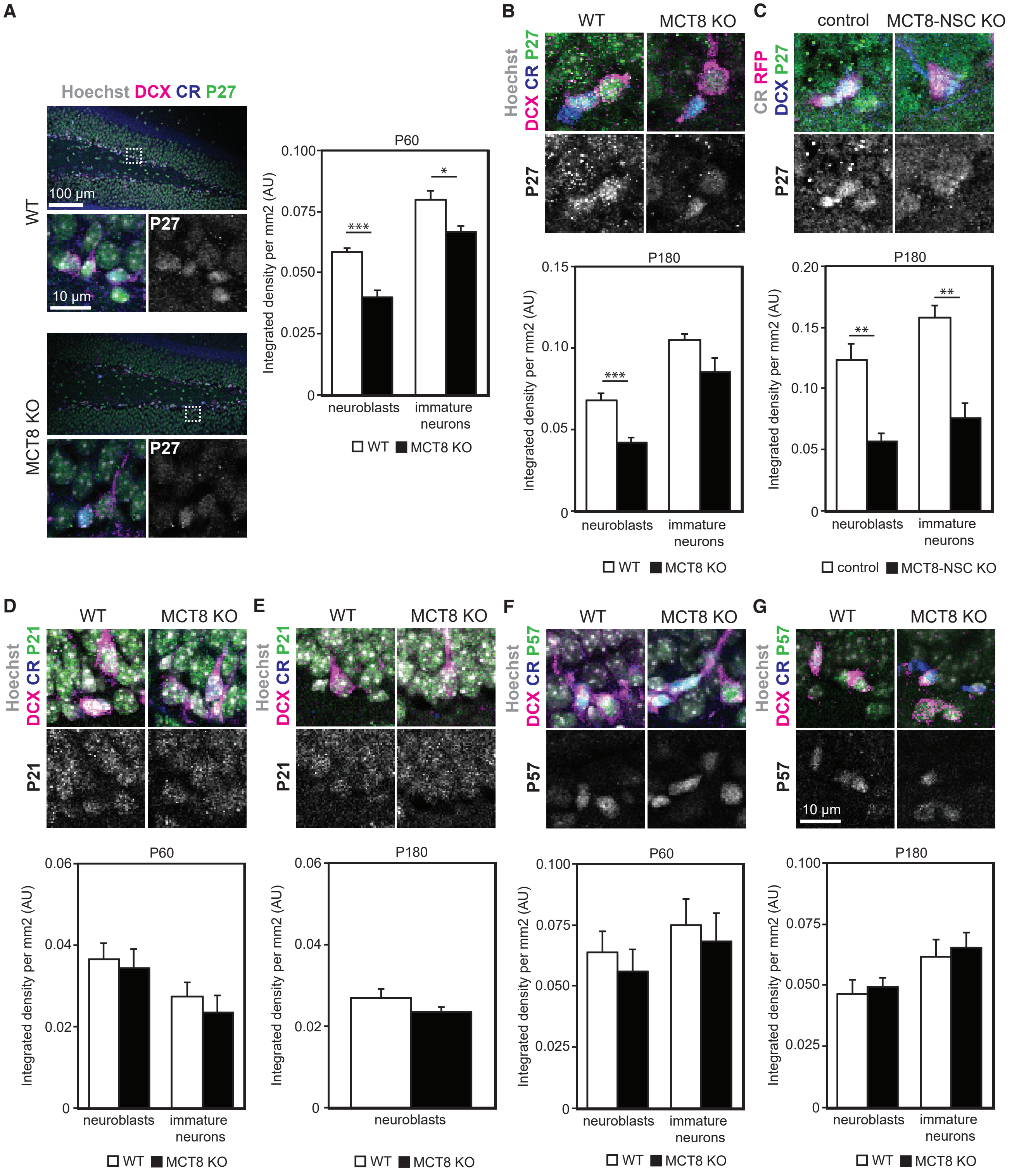

Figure 7. Cell-Cycle Inhibitor Expression Is Altered in MCT8 Deficiency

(A) Representative overview images and magnified views of P27KIP1 (green) staining in DCX+ (magenta) type 2b progenitors/NBs and DCX+/CR+ (blue) INs at 2 months of age. Normalized nuclear P27 fluorescent signal intensities were quantified.

(B) P27 immunoreactivity in type 2b progenitors/NBs and INs was measured at 6 months of age.

(legend continued on next page)

the earlier DCX expression discussed above), so explaining the smaller reduction in NBs at 6 months in these mice. Clearly, any of the neighboring glial and vascular cell types could contribute to this non-cell-autonomous effect. We hypothesize, however, that MCT8-deficient astrocytes contribute significantly through the previously described effects on TH-regulated components of the NOTCH or WNT signaling pathways (Morte et al., 2018).

The role of MCT8 at the differentiation stage of neurogenesis is of particular relevance to the pathophysiology of Allan-Herndon-Dudley syndrome (AHDS), a severe form of psychomotor retardation caused by inactivating mutations in MCT8 (Dumitrescu et al., 2004; Friesema et al., 2004; Schwartz et al., 2005). AHDS-like symptoms could be replicated only by simultaneous inactivation of MCT8 and another TH transporter, OATP1C1, in mice (Mayerl et al., 2014), from which we presumed that impaired TH transport across the BBB and/or BCSFB is the major abnormality driving the disease phenotype. Our results that MCT8 loss results in cell-autonomous effects in NBs, however, suggests that a direct effect on the formation of newly born GCNs may also contribute to the phenotype.

MCT8 has recently been implicated in corticogenesis in the chicken optic tectum (Vancamp et al., 2017), where knockdown resulted in a reduced progenitor pool and diminished neurogenesis. Although we also observed reduced neurogenesis we found, in contrast to Vancamp et al., that MCT8 was critical for later stages of the adult hippocampal program in the mouse, i.e., the differentiation step from NBs to INs, and not for the regulation of progenitor proliferation and pool size. This emphasizes that the mechanisms by which TH signaling influences neurogenic processes vary between niches. Similarly, a different function of the TH signal is documented in a third niche, the adult SVZ, where, in contrast to the SGZ T3/TRα1, signaling is involved in repressing NSC pluripotency and determining the progenitor pool size (Remaud et al., 2014).

The organotypic hippocampal slice culture system that we established is likely to have significant utility. Our protocol that allows adult slices to be maintained for up to 3 weeks enables examination of all stages of adult neurogenesis and cell-fate monitoring of EdU+ cells. We used the technique to show that application of the MCT8-specific inhibitor silychristin compromised generation of EdU+/NEUN+ neurons after 3 weeks in culture without effects on EdU incorporation at earlier stages. This is in line with earlier *in vivo* studies of hypothyroid rodents that reported either no effect or only a slightly reduced progenitor proliferation of in the SGZ, whereas formation of new neurons was impaired (Ambrogini et al., 2005; Desouza et al., 2005; Montero-Pedrazuela et al., 2006). In comparison, application of the LAT inhibitor BCH or the deiodinase inhibitor iopanoic acid did not alter neurogenesis in hippocampal slices. These findings underscore a prominent gate-keeper role of MCT8 in NBs and substantiate the view that, in the SGZ, TH predominantly acts on post-mitotic progenitors (Remaud et al., 2014).

To define a mechanism by which MCT8 in NBs is required for proper differentiation we investigated the expression of cell-cycle/CDK inhibitors P21CIP1, P27KIP1, and P57KIP2. In line with recent work (Horster et al., 2017) we found pronounced P27 expression in SGZ NBs and INs of WT mice, whereas significantly lower P27 protein and mRNA levels were detected in MCT8 KO and MCT8-NSC KO mice. Based on that and reports showing *p27* as a TH target gene (Garcia-Silva et al., 2002; Holsberger et al., 2003), we speculate that absence of MCT8 in NBs causes TH deficiency within the cells, which in turn reduces the expression of P27 and inhibits differentiation. Consistent with this, P27-deficient mice have more proliferating cells in the SGZ, reduced levels of neurogenesis, and specific cognitive impairments (Horster et al., 2017).

Our demonstration that, in the CNS, loss of MCT8 causes both cell-autonomous and non-cell-autonomous effects on neurogenesis will inform potential treatment strategies for AHDS where, in addition to any transport impairments across the BBB, effects of MCT8 loss in CNS cell populations will need to be addressed. Our findings also have important implications for therapeutic approaches addressing cognitive decline resulting from compromised hippocampal neurogenesis, where selective targeting of the cell-autonomous functions of TH signaling may allow enhanced neuronal differentiation without the systemic effects of increased TH action.

EXPERIMENTAL PROCEDURES

Animals

MCT8 KO mice obtained from Deltagen were generated, bred, and genotyped as described previously (Trajkovic et al., 2007). *Mct8*fl mice obtained from the KOMP repository (*Slc16a2*$^{tm1a(KOMP)Wtsi}$) were generated and genotyped as reported before (Mayerl et al.,

(C) Sections from 6-month-old MCT8-NSC KO and control brains were stained for P27KIP1 (green), DCX (blue), and CR (gray). RFP fluorescence is shown in magenta. Magnified views depict CR−/RFP+/DCX+ cells. Normalized P27 signal intensities were compared.
(D and E) P21 (green) was analyzed at 2 months (D) and 6 months of age (E).
(F and G) P57 (green) fluorescence intensities were assessed at 2 months (F) and 6 months (G) of age. Hoechst 33258-positive nuclei are shown in gray, DCX in magenta, and CR in blue. n = 6 (A, B, and D–G) and n = 4–5 (C) mice per genotype. Group means + SEM are shown.*, $p < 0.05$; **, $p < 0.01$; ***, $p < 0.001$, unpaired two-tailed Student's t test.

2018). *Mct8*+/ KO and *Mct8*+/fl females were bred with males (C57BL/6) carrying a tamoxifen-inducible Cre recombinase driven by the *Nestin* promoter (Lagace et al., 2007) and a *Cre* reporter allele consisting of a *loxP*-flanked STOP cassette preventing transcription of a CAG promotor-driven *tdTomato* construct (Madisen et al., 2010) purchased from Jackson Laboratories (C57BL/6-Tg*[Nes-cre/ERT2]KEisc/J*, Jax stock no. 016261 and *B6.Cg-Gt(ROSA) 26Sor*$^{tm9(CAG\text{-}tdTomato)Hze}$, Jax stock no. 007909). *Cre* and *tdTomato* (hereafter *Rfp*) transgenes were detected as described (Lagace et al., 2007; Madisen et al., 2010). At the age of 4–5 weeks, tamoxifen (180 mg/kg; Sigma-Aldrich) was administered to *Mct8*+/y, *Mct8* KO/y, and *Mct8*fl/y male mice (note that the *Mct8* gene is located on the X chromosome) harboring both transgenes by oral gavage for 5 consecutive days and animals were kept for 5 months. For EdU-labeling studies, mice (aged 2 or 6 months) were injected intraperitoneally with 100 μL EdU (10 mg/mL; Thermo Fisher Scientific) in PBS 3 or 28 days before sacrifice. Six- to 8-week-old mice for hippocampal slice cultures were injected twice with EdU as above 4 and 2 h before sacrifice.

Mice were kept at constant temperature (22°C) on a 12-h light/dark cycle and provided with standard chow and water *ad libitum*. Animals used for FACS studies were sacrificed by cervical dislocation at 2 months of age. For immunofluorescence studies mice were transcardially perfused with 4% paraformaldehyde (PFA). Brains were cryo-protected with 30% sucrose, snap frozen in isopentane on dry ice and kept at −80°C. Mice designated for hippocampal slice culture were exposed to rising concentrations of CO_2 and brains were isolated rapidly. For all studies, male mice have been used.

FACS

For one run, brains of eight C57BL/6N WT mice were isolated, stored in chilled Hibernate A (Thermo Fisher Scientific), and dentate gyri were micro-dissected (Babu et al., 2011). Tissue was pooled and processed as described (Guez-Barber et al., 2012) and as summarized in the Supplemental Information.

Before fixation, cells were re-suspended in Hibernate A, incubated for 15 min with a fixable live/dead cell stain (LIVE/DEAD Fixable Violet Dead Cell Stain Kit; Life Technologies, 1:1,000) at 4°C and pelleted by centrifugation at 4,000 rpm for 4 min. For fixation, cells were re-suspended in 1 mL chilled Hibernate A, 3 mL of ice-cold 100% ethanol (molecular grade; Sigma-Aldrich) was added, and cells were fixed in this 75% ethanol solution at 4°C for 20 min (Diez et al., 1999). To increase mRNA yield and quality, all solutions used after this step were treated overnight with 1:1,000 diethyl pyrocarbonate (Sigma-Aldrich) (Diez et al., 1999). After fixation, cells were pelleted as above and washed in 1 mL chilled PBS containing 0.1% saponin (Sigma-Aldrich), 0.2% BSA (Sigma-Aldrich), and 1:100 RNase inhibitor (RNaseOUT, Life Technologies) (Hrvatin et al., 2014). Staining procedures are detailed in the Supplemental Information.

Hippocampal neurogenic populations were sorted with a FACSAria II (BD Bioscience) into chilled RNase-free tubes containing 100 μL FACS buffer. All marker-negative cells were collected separately for RNA quality determination. If the final volume per tube exceeded 300 μL, cells were pelleted by centrifugation for 10 min at 13,200 rpm and 4°C. Cells were frozen on dry ice and stored at −80°C.

Adult Hippocampal Slice Culture

Mouse brains were isolated and transferred into chilled dissection buffer (Hibernate A with 2% B27 supplement [Life Technologies], 2 mM L-glutamine and 1% penicillin/streptomycin) on ice as described (Kim et al., 2013). Tissue was sectioned as published previously (Kleine Borgmann et al., 2013) in chilled dissection buffer using a vibratome. Sections (300 μm) were stored in dissection buffer on ice and transferred onto Millicell inserts (Millipore). Organotypic slices were cultured at 37°C and 5% CO_2 in a serum-free medium (Kim et al., 2013) (Neurobasal A [Life Technologies] containing 2% B27 supplement, 2 mM L-glutamine, 1% penicillin/streptomycin, and 80 μM indomethacin [Sigma-Aldrich]). During the entire culture period slices were exposed to 25 μM silychristin (Sigma-Aldrich), 10 μM iopanoic acid (Sigma-Aldrich), 1 mM BCH (R&D Systems), or respective volumes of the solvents DMSO or culture medium as control. Culture medium was replaced every other day and slices were fixed for 1 h in 4% PFA.

Immunofluorescence Studies, Quantification, and RT-PCR

Procedures are described in the Supplemental Information.

qPCR

Total RNA from FAC-sorted hippocampal populations and respective controls was isolated using the RNEasy Micro Kit (QIAGEN). RNA quality was assessed in controls on a high sensitivity screen tape (Agilent Technologies). At least 100,000 cells from control sorts were subjected to RIN value assessment. Samples were only processed further if RIN ≥ 7. Two rounds of RNA amplification were conducted using the ExpressArt C&E PICO RNA Amplification Kit (AMS Biotechnology) following the manufacturer's instructions. RNA concentration was analyzed with an RNA screentape (Agilent Technologies). If necessary, a third round of RNA amplification was performed and quantity was measured as before. A total of 250 ng of RNA was subjected to cDNA synthesis using the SuperScript First-Strand Synthesis System (Invitrogen). Quantitative Real-Time PCR (qPCR) was performed using the QuantiFast SYBR Green PCR Kit (QIAGEN) and the LightCycler 480 system (Roche). Further information can be found in the Supplemental Information material.

Statistics

All data represent mean + SEM. In slice culture experiments, to compare WT versus MCT8 KO animals and control versus MCT8-NSC KO mice statistical significance between groups was determined by unpaired two-tailed Student's t test. Differences were considered significant when $p < 0.05$ and marked as follows $^*p < 0.05$, $^{**}p < 0.01$, $^{***}p < 0.001$.

Study Approval

All studies were executed in compliance with UK Home Office regulations and local guidelines by The University of Edinburgh.

SUPPLEMENTAL INFORMATION

Supplemental Information can be found online at https://doi.org/10.1016/j.stemcr.2020.03.014.

AUTHOR CONTRIBUTIONS

S.M. devised, conducted, and analyzed the experiments. S.M. and C.ff-C. interpreted the results. H.H. provided *Mct8* KO and *Mct8*fl mice as well as valuable expertise on TH signaling. S.M., H.H., and C.ff-C. wrote the manuscript.

ACKNOWLEDGMENTS

This work was supported by grants from the DFG to S.M. (MA7212/2-1) and H.H. (HE3418/8-1 within the SPP1629), and by a Wellcome Trust Senior Investigator award (104783/Z/14/Z) and funding from the BBSRC (BB/L00402X/1) to C.ff-C. We thank SCRM animal facility staff (Luke McPhee, Chris Wilson, Lorraine McNeil, John Agnew, and Jamie Kelly) for their excellent help. We are also grateful to Fiona Rossi and Claire Cryer (SCRM FACS facility) and Bertrand Vernay (SCRM imaging facility) for support.

Received: October 12, 2019
Revised: March 14, 2020
Accepted: March 17, 2020
Published: April 16, 2020

REFERENCES

Ambrogini, P., Cuppini, R., Ferri, P., Mancini, C., Ciaroni, S., Voci, A., Gerdoni, E., and Gallo, G. (2005). Thyroid hormones affect neurogenesis in the dentate gyrus of adult rat. Neuroendocrinology *81*, 244–253.

Astapova, I., and Hollenberg, A.N. (2013). The in vivo role of nuclear receptor corepressors in thyroid hormone action. Biochim. Biophys. Acta *1830*, 3876–3881.

Babu, H., Claasen, J.H., Kannan, S., Rünker, A.E., Palmer, T., and Kempermann, G. (2011). A protocol for isolation and enriched monolayer cultivation of neural precursor cells from mouse dentate gyrus. Front. Neurosci. *5*, 89.

Beckervordersandforth, R., Zhang, C.L., and Lie, D.C. (2015). Transcription-factor-dependent control of adult hippocampal neurogenesis. Cold Spring Harb. Perspect. Biol. *7*, a018879.

Ben Abdallah, N.M., Slomianka, L., Vyssotski, A.L., and Lipp, H.P. (2010). Early age-related changes in adult hippocampal neurogenesis in C57 mice. Neurobiol. Aging *31*, 151–161.

Bernal, J., Guadano-Ferraz, A., and Morte, B. (2015). Thyroid hormone transporters—functions and clinical implications. Nat. Rev. Endocrinol. *11*, 406–417.

Bianco, A.C., Salvatore, D., Gereben, B., Berry, M.J., and Larsen, P.R. (2002). Biochemistry, cellular and molecular biology, and physiological roles of the iodothyronine selenodeiodinases. Endocr. Rev. *23*, 38–89.

Ceballos, A., Belinchon, M.M., Sanchez-Mendoza, E., Grijota-Martinez, C., Dumitrescu, A.M., Refetoff, S., Morte, B., and Bernal, J. (2009). Importance of monocarboxylate transporter 8 for the blood-brain barrier-dependent availability of 3,5,3′-triiodo-L-thyronine. Endocrinology *150*, 2491–2496.

Cooke, G.E., Mullally, S., Correia, N., O'Mara, S.M., and Gibney, J. (2014). Hippocampal volume is decreased in adults with hypothyroidism. Thyroid *24*, 433–440.

Correia, N., Mullally, S., Cooke, G., Tun, T.K., Phelan, N., Feeney, J., Fitzgibbon, M., Boran, G., O'Mara, S., and Gibney, J. (2009). Evidence for a specific defect in hippocampal memory in overt and subclinical hypothyroidism. J. Clin. Endocrinol. Metab. *94*, 3789–3797.

Dentice, M., Marsili, A., Zavacki, A., Larsen, P.R., and Salvatore, D. (2013). The deiodinases and the control of intracellular thyroid hormone signaling during cellular differentiation. Biochim. Biophys. Acta *1830*, 3937–3945.

Desouza, L.A., Ladiwala, U., Daniel, S.M., Agashe, S., Vaidya, R.A., and Vaidya, V.A. (2005). Thyroid hormone regulates hippocampal neurogenesis in the adult rat brain. Mol. Cell. Neurosci. *29*, 414–426.

Diez, C., Bertsch, G., and Simm, A. (1999). Isolation of full-size mRNA from cells sorted by flow cytometry. J. Biochem. Biophys. Methods *40*, 69–80.

Dumitrescu, A.M., Liao, X.H., Best, T.B., Brockmann, K., and Refetoff, S. (2004). A novel syndrome combining thyroid and neurological abnormalities is associated with mutations in a monocarboxylate transporter gene. Am. J. Hum. Genet. *74*, 168–175.

Flamant, F., and Gauthier, K. (2013). Thyroid hormone receptors: the challenge of elucidating isotype-specific functions and cell-specific response. Biochim. Biophys. Acta *1830*, 3900–3907.

Friesema, E.C., Grueters, A., Biebermann, H., Krude, H., von Moers, A., Reeser, M., Barrett, T.G., Mancilla, E.E., Svensson, J., Kester, M.H., et al. (2004). Association between mutations in a thyroid hormone transporter and severe X-linked psychomotor retardation. Lancet *364*, 1435–1437.

Garcia-Silva, S., Perez-Juste, G., and Aranda, A. (2002). Cell cycle control by the thyroid hormone in neuroblastoma cells. Toxicology *181-182*, 179–182.

Gerlach, J., Donkels, C., Münzner, G., and Haas, C.A. (2016). Persistent gliosis interferes with neurogenesis in organotypic hippocampal slice cultures. Front. Cell. Neurosci. *10*, 131.

Guez-Barber, D., Fanous, S., Harvey, B.K., Zhang, Y., Lehrmann, E., Becker, K.G., Picciotto, M.R., and Hope, B.T. (2012). FACS purification of immunolabeled cell types from adult rat brain. J. Neurosci. Methods *203*, 10–18.

Heuer, H., and Visser, T.J. (2013). The pathophysiological consequences of thyroid hormone transporter deficiencies: insights from mouse models. Biochim. Biophys. Acta *1830*, 3974–3978.

Holsberger, D.R., Jirawatnotai, S., Kiyokawa, H., and Cooke, P.S. (2003). Thyroid hormone regulates the cell cycle inhibitor p27Kip1 in postnatal murine Sertoli cells. Endocrinology *144*, 3732–3738.

Horster, H., Garthe, A., Walker, T.L., Ichwan, M., Steiner, B., Khan, M.A., Lie, D.C., Nicola, Z., Ramirez-Rodriguez, G., and Kempermann, G. (2017). p27kip1 is required for functionally relevant adult hippocampal neurogenesis in mice. Stem Cells *35*, 787–799.

Hrvatin, S., Deng, F., O'Donnell, C.W., Gifford, D.K., and Melton, D.A. (2014). MARIS: method for analyzing RNA following intracellular sorting. PLoS One *9*, e89459.

Ittermann, T., Wittfeld, K., Nauck, M., Bulow, R., Hosten, N., Volzke, H., and Grabe, H.J. (2018). High thyrotropin is associated

with reduced hippocampal volume in a population-based study from Germany. Thyroid *28*, 1434–1442.

Johannes, J., Jayarama-Naidu, R., Meyer, F., Wirth, E.K., Schweizer, U., Schomburg, L., Kohrle, J., and Renko, K. (2016). Silychristin, a flavonolignan derived from the milk thistle, is a potent inhibitor of the thyroid hormone transporter MCT8. Endocrinology *157*, 1694–1701.

Kapoor, R., Desouza, L.A., Nanavaty, I.N., Kernie, S.G., and Vaidya, V.A. (2012). Thyroid hormone accelerates the differentiation of adult hippocampal progenitors. J. Neuroendocrinol. *24*, 1259–1271.

Kapoor, R., Ghosh, H., Nordstrom, K., Vennstrom, B., and Vaidya, V.A. (2011). Loss of thyroid hormone receptor beta is associated with increased progenitor proliferation and NeuroD positive cell number in the adult hippocampus. Neurosci. Lett. *487*, 199–203.

Kapoor, R., van Hogerlinden, M., Wallis, K., Ghosh, H., Nordstrom, K., Vennstrom, B., and Vaidya, V.A. (2010). Unliganded thyroid hormone receptor alpha1 impairs adult hippocampal neurogenesis. FASEB J. *24*, 4793–4805.

Kempermann, G., Jessberger, S., Steiner, B., and Kronenberg, G. (2004). Milestones of neuronal development in the adult hippocampus. Trends Neurosci. *27*, 447–452.

Kim, H., Kim, E., Park, M., Lee, E., and Namkoong, K. (2013). Organotypic hippocampal slice culture from the adult mouse brain: a versatile tool for translational neuropsychopharmacology. Prog. Neuropsychopharmacol. Biol. Psychiatry *41*, 36–43.

Kleine Borgmann, F.B., Bracko, O., and Jessberger, S. (2013). Imaging neurite development of adult-born granule cells. Development *140*, 2823–2827.

Koenig, R.J., Lazar, M.A., Hodin, R.A., Brent, G.A., Larsen, P.R., Chin, W.W., and Moore, D.D. (1989). Inhibition of thyroid hormone action by a non-hormone binding c-erbA protein generated by alternative mRNA splicing. Nature *337*, 659–661.

Kuhn, H.G., Toda, T., and Gage, F.H. (2018). Adult hippocampal neurogenesis: a coming-of-age story. J. Neurosci. *38*, 10401–10410.

Lagace, D.C., Whitman, M.C., Noonan, M.A., Ables, J.L., DeCarolis, N.A., Arguello, A.A., Donovan, M.H., Fischer, S.J., Farnbauch, L.A., Beech, R.D., et al. (2007). Dynamic contribution of nestin-expressing stem cells to adult neurogenesis. J. Neurosci. *27*, 12623–12629.

Madisen, L., Zwingman, T.A., Sunkin, S.M., Oh, S.W., Zariwala, H.A., Gu, H., Ng, L.L., Palmiter, R.D., Hawrylycz, M.J., Jones, A.R., et al. (2010). A robust and high-throughput Cre reporting and characterization system for the whole mouse brain. Nat. Neurosci. *13*, 133–140.

Mayerl, S., Muller, J., Bauer, R., Richert, S., Kassmann, C.M., Darras, V.M., Buder, K., Boelen, A., Visser, T.J., and Heuer, H. (2014). Transporters MCT8 and OATP1C1 maintain murine brain thyroid hormone homeostasis. J. Clin. Invest. *124*, 1987–1999.

Mayerl, S., Schmidt, M., Doycheva, D., Darras, V.M., Huttner, S.S., Boelen, A., Visser, T.J., Kaether, C., Heuer, H., and von Maltzahn, J. (2018). Thyroid hormone transporters MCT8 and OATP1C1 control skeletal muscle regeneration. Stem Cell Reports *10*, 1959–1974.

Melo-Salas, M.S., Perez-Dominguez, M., and Zepeda, A. (2018). Systemic inflammation impairs proliferation of hippocampal type 2 intermediate precursor cells. Cell. Mol. Neurobiol. *38*, 1517–1528.

Miller, K.J., Parsons, T.D., Whybrow, P.C., van Herle, K., Rasgon, N., van Herle, A., Martinez, D., Silverman, D.H., and Bauer, M. (2006). Memory improvement with treatment of hypothyroidism. Int. J. Neurosci. *116*, 895–906.

Montero-Pedrazuela, A., Venero, C., Lavado-Autric, R., Fernandez-Lamo, I., Garcia-Verdugo, J.M., Bernal, J., and Guadano-Ferraz, A. (2006). Modulation of adult hippocampal neurogenesis by thyroid hormones: implications in depressive-like behavior. Mol. Psychiatry *11*, 361–371.

Morte, B., Gil-Ibanez, P., and Bernal, J. (2018). Regulation of gene expression by thyroid hormone in primary astrocytes: factors influencing the genomic response. Endocrinology *159*, 2083–2092.

Osterweil, D., Syndulko, K., Cohen, S.N., Pettler-Jennings, P.D., Hershman, J.M., Cummings, J.L., Tourtellotte, W.W., and Solomon, D.H. (1992). Cognitive function in non-demented older adults with hypothyroidism. J. Am. Geriatr. Soc. *40*, 325–335.

Remaud, S., Gothie, J.D., Morvan-Dubois, G., and Demeneix, B.A. (2014). Thyroid hormone signaling and adult neurogenesis in mammals. Front. Endocrinol. (Lausanne) *5*, 62.

Ritchie, J.W., Peter, G.J., Shi, Y.B., and Taylor, P.M. (1999). Thyroid hormone transport by 4F2hc-IU12 heterodimers expressed in *Xenopus* oocytes. J. Endocrinol. *163*, R5–R9.

Ritchie, J.W., Shi, Y.B., Hayashi, Y., Baird, F.E., Muchekehu, R.W., Christie, G.R., and Taylor, P.M. (2003). A role for thyroid hormone transporters in transcriptional regulation by thyroid hormone receptors. Mol. Endocrinol. *17*, 653–661.

Schwartz, C.E., May, M.M., Carpenter, N.J., Rogers, R.C., Martin, J., Bialer, M.G., Ward, J., Sanabria, J., Marsa, S., Lewis, J.A., et al. (2005). Allan-Herndon-Dudley syndrome and the monocarboxylate transporter 8 (MCT8) gene. Am. J. Hum. Genet. *77*, 41–53.

Suzuki, S., Mori, J., and Hashizume, K. (2007). mu-Crystallin, a NADPH-dependent T(3)-binding protein in cytosol. Trends Endocrinol. Metab. *18*, 286–289.

Trajkovic, M., Visser, T.J., Mittag, J., Horn, S., Lukas, J., Darras, V.M., Raivich, G., Bauer, K., and Heuer, H. (2007). Abnormal thyroid hormone metabolism in mice lacking the monocarboxylate transporter 8. J. Clin. Invest. *117*, 627–635.

Vancamp, P., Deprez, M.A., Remmerie, M., and Darras, V.M. (2017). Deficiency of the thyroid hormone transporter monocarboxylate transporter 8 in neural progenitors impairs cellular processes crucial for early corticogenesis. J. Neurosci. *37*, 11616–11631.

Stem Cell Reports, Volume *14*

Supplemental Information

Hippocampal Neurogenesis Requires Cell-Autonomous Thyroid Hormone Signaling

Steffen Mayerl, Heike Heuer, and Charles ffrench-Constant

Fig.S1

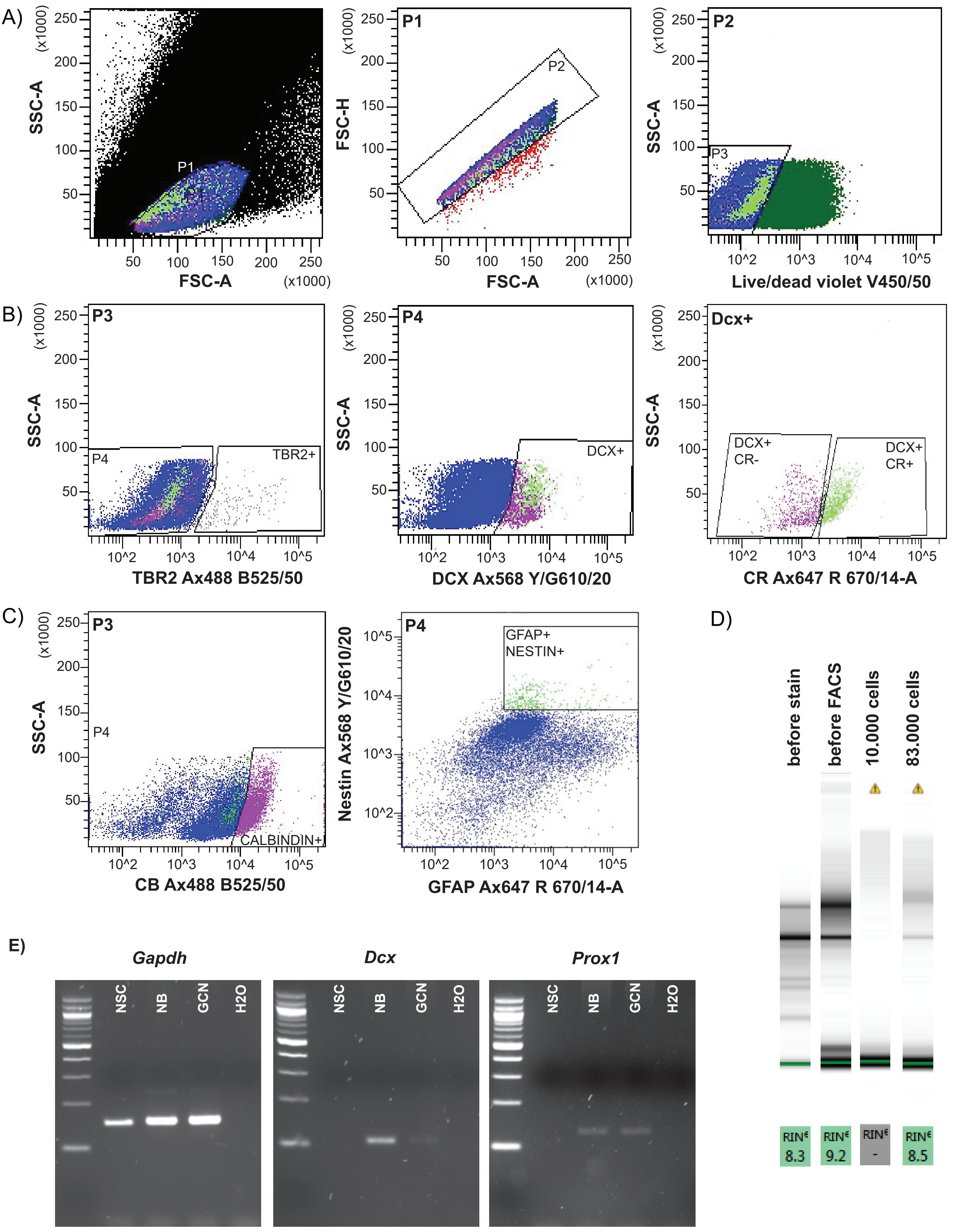
A)
SSC-A
FSC-A
(x1000)
P1
FSC-H
P2
Live/dead violet V450/50
P3
B)
TBR2 Ax488 B525/50
P4
TBR2+
DCX Ax568 Y/G610/20
DCX+
Dcx+
DCX+
CR-
DCX+
CR+
CR Ax647 R 670/14-A
C)
CALBINDIN+
CB Ax488 B525/50
Nestin Ax568 Y/G610/20
GFAP+
NESTIN+
GFAP Ax647 R 670/14-A
D)
before stain
before FACS
10.000 cells
83.000 cells
RIN 8.3
RIN 9.2
RIN -
RIN 8.5
E)
Gapdh
Dcx
Prox1
NSC
NB
GCN
H2O

Fig.S2

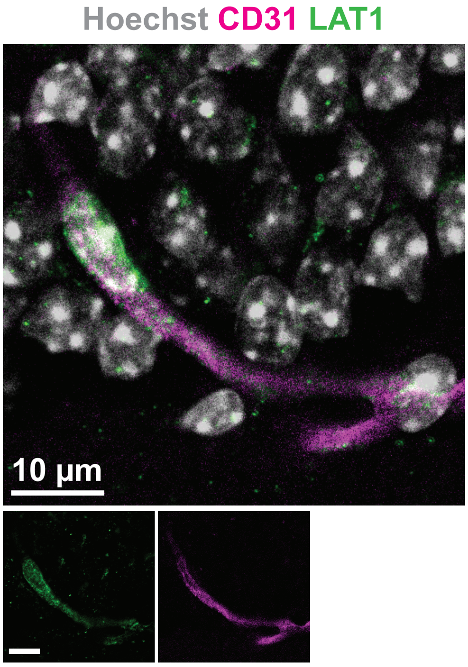

Fig.S3

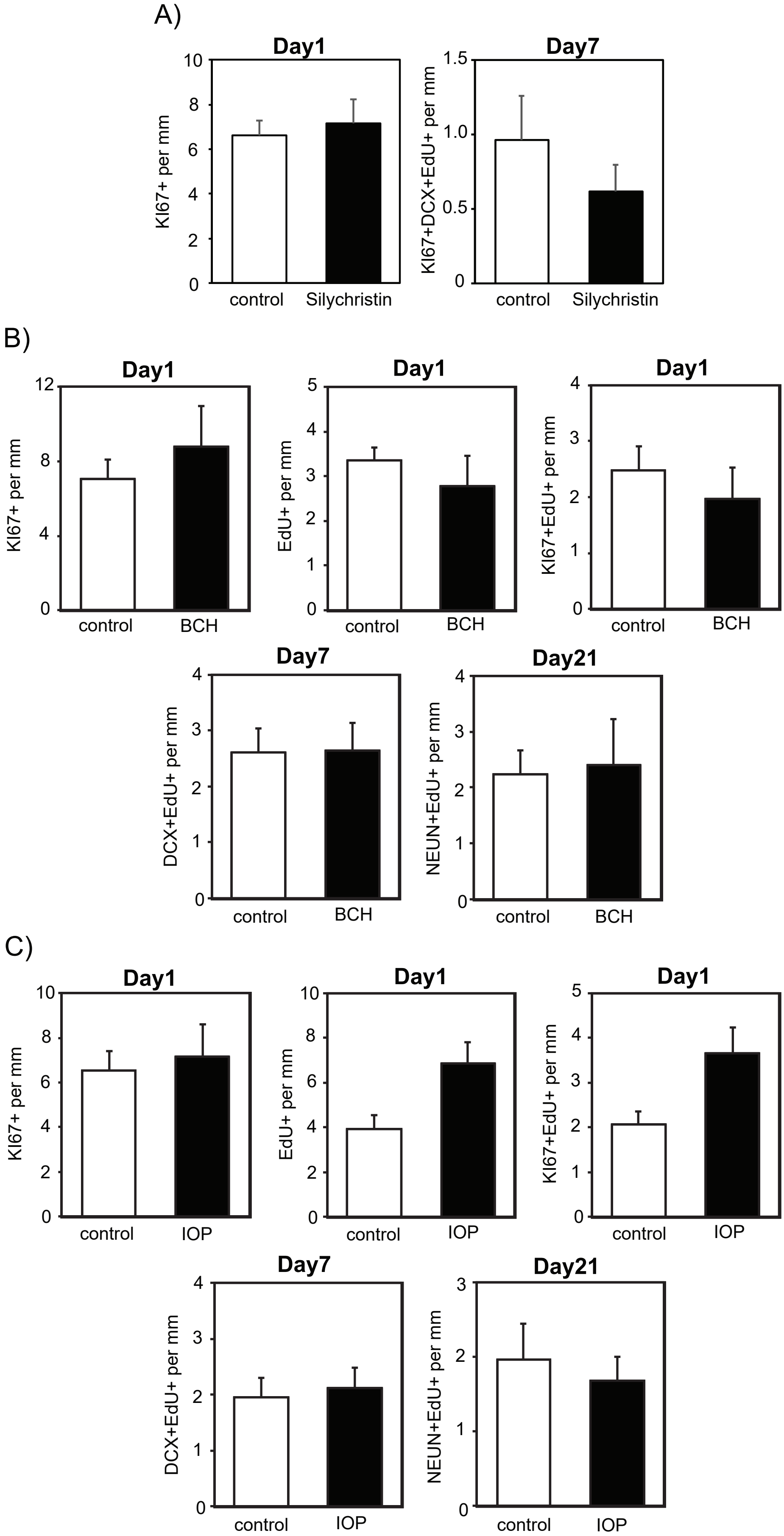
A)
Day1
Day7
KI67+ per mm
KI67+DCX+EdU+ per mm
control
Silychristin
B)
Day1
Day7
Day21
KI67+ per mm
EdU+ per mm
KI67+EdU+ per mm
DCX+EdU+ per mm
NEUN+EdU+ per mm
control
BCH
C)
Day1
Day7
Day21
KI67+ per mm
EdU+ per mm
KI67+EdU+ per mm
DCX+EdU+ per mm
NEUN+EdU+ per mm
control
IOP

Fig.S4

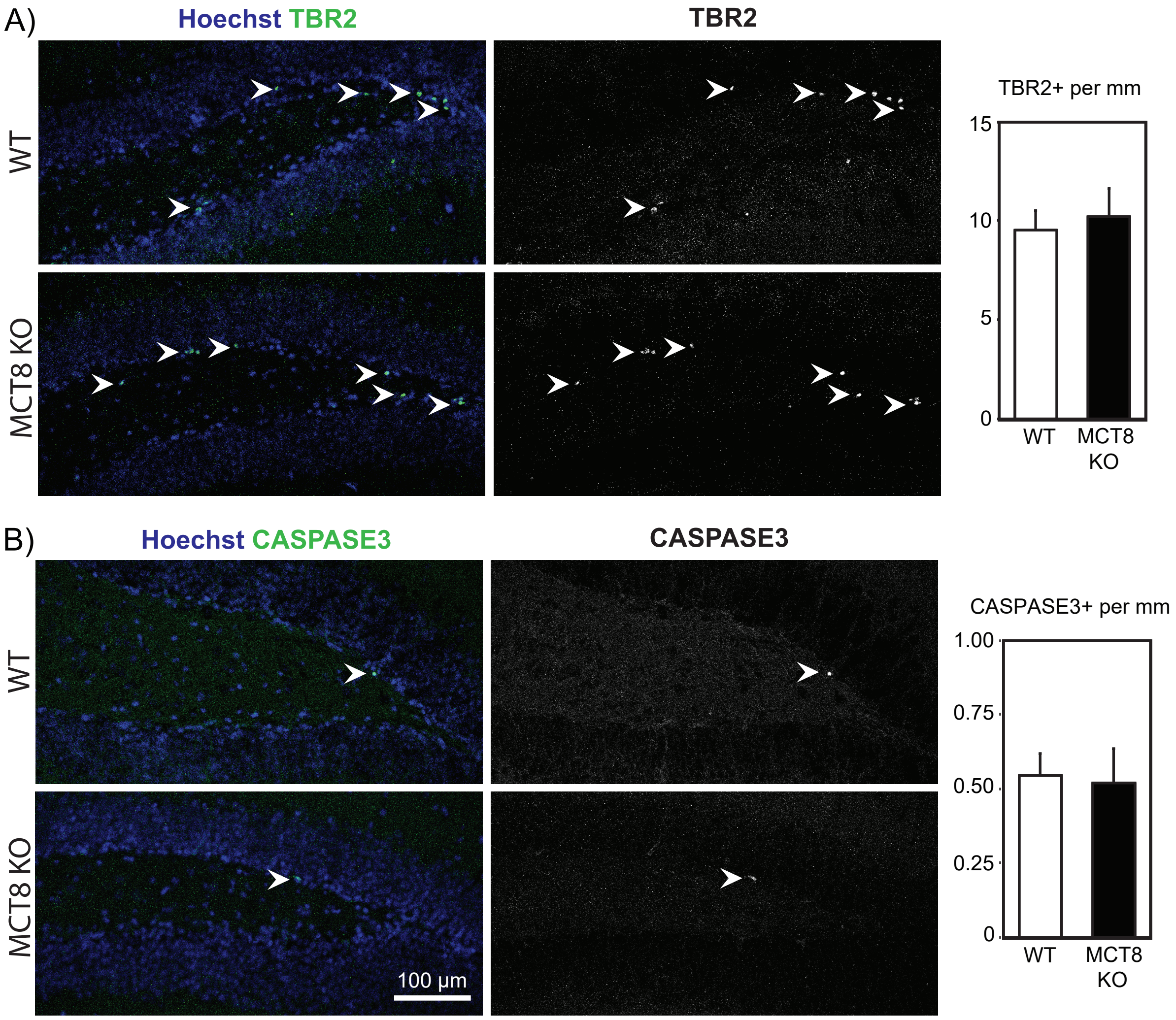
A)
Hoechst TBR2
TBR2
WT
MCT8 KO
TBR2+ per mm
15
10
5
0
WT
MCT8 KO
B)
Hoechst CASPASE3
CASPASE3
WT
MCT8 KO
100 µm
CASPASE3+ per mm
1.00
0.75
0.50
0.25
0
WT
MCT8 KO

Fig.S5

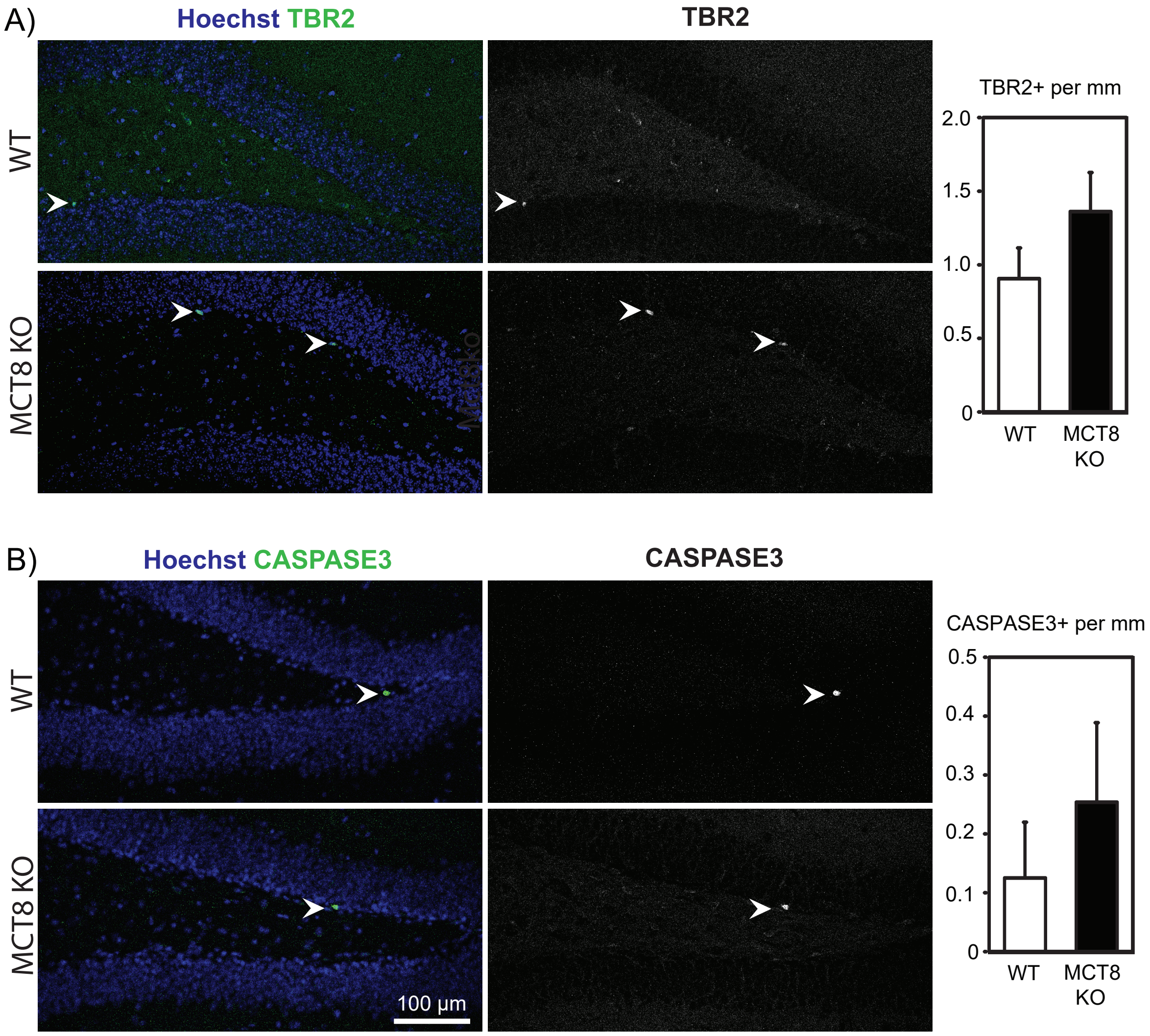

A)
Hoechst TBR2
TBR2
WT
MCT8 KO
TBR2+ per mm
2.0
1.5
1.0
0.5
0
WT
MCT8 KO
B)
Hoechst CASPASE3
CASPASE3
WT
MCT8 KO
100 µm
CASPASE3+ per mm
0.5
0.4
0.3
0.2
0.1
0
WT
MCT8 KO

Fig.S6

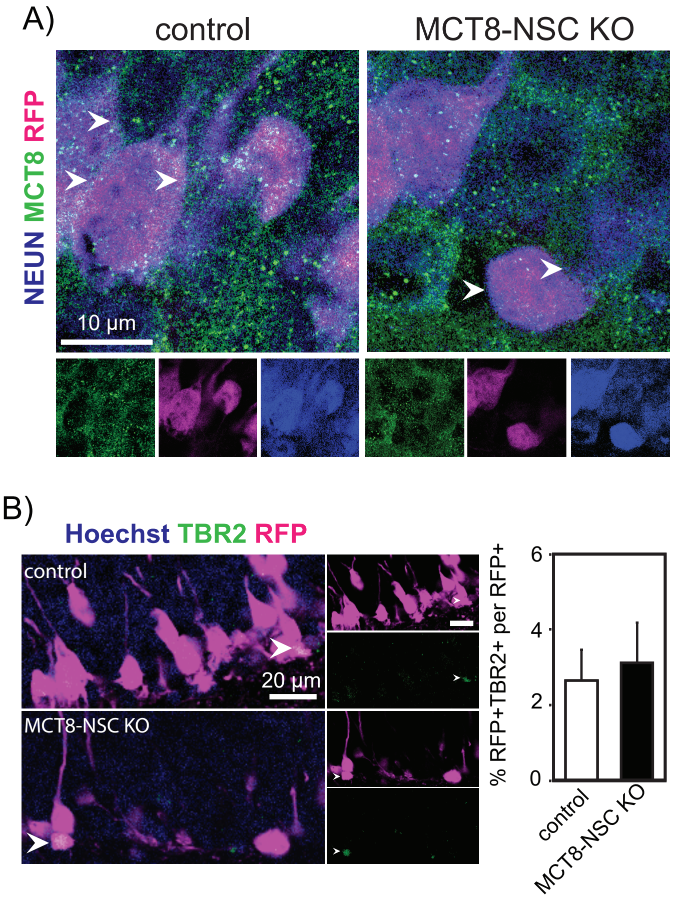
A)
control
MCT8-NSC KO
NEUN MCT8 RFP
10 µm
B)
Hoechst TBR2 RFP
control
20 µm
MCT8-NSC KO
% RFP+TBR2+ per RFP+
6
4
2
0
control
MCT8-NSC KO

Fig.S7

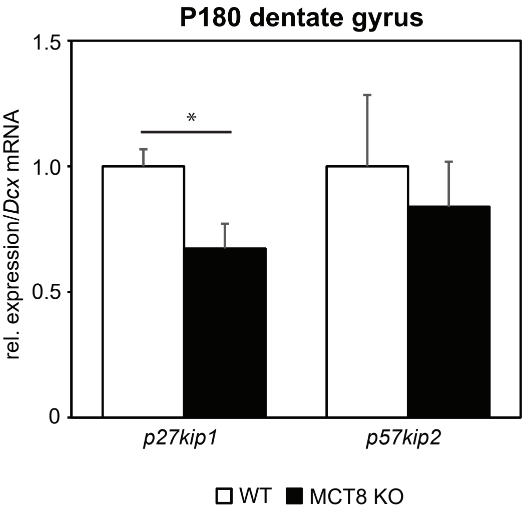
P180 dentate gyrus
1.5
1.0
0.5
0
rel. expression/Dcx mRNA
*
p27kip1
p57kip2
WT
MCT8 KO

Supplementary figure legends:

Fig.S1: FACsorting strategy and RNA integrity control. Related to Fig. 1

Micro-dissected dentate gyri were subjected to flow cytometry and different neurogenic/neuronal populations were sorted according to their expression of intracellular markers. A) Flow cytometry strategy is shown to separate cells from debris (P1), single cells from cell clusters (P2) and living cells within the P2 population that were identified by low levels of a fixable live/dead cell stain (P3). B) Samples were stained for TBR2(AxF488), DCX(AxF568) and CR(AxF647) and TBR2+ (TAP), DCX+/CR- (NB) as well as DCX+/CR+ (IN) cell populations were separated. TBR2-/DCX- cells were collected as control for RIN value determination. C) Samples were stained for CB(AxF488), NESTIN(AxF568) and GFAP(AxF647). First, CB+ cells were separated (GCN) followed by sorting into NESTIN+/GFAP+ (NSC) and non-NSCs used for RIN value determination. D) RIN values were assessed after indicated steps. After FACS, higher cell numbers (≥83.000) were needed to determine reliably RIN values. E) RNA was isolated, amplified and reversely transcribed from different samples and subjected to RT-PCR. Expression pattern of the house keeping gene *Gapdh* and neurogenic markers *Dcx* and *Prox1* were evaluated. NSC-neural stem cells; TAP-transiently amplifying progenitors; NB-neuroblasts; IN-immature neurons; GCN-granule cell neurons.

Fig.S2: Expression of TH signaling components in the DG. Related to Fig. 2

Coronal forebrain cryosections were incubated with antibodies against LAT1 (in green) and the endothelial cell marker CD31/PECAM (in magenta). Nuclei stained with Hoechst33258 appear gray.

Fig.S3: BCH and IOP do not alter hippocampal neurogenesis ex vivo. Related to Fig. 3

Adult mouse brain slices were kept in culture for up to 21 days in the presence of inhibitors of TH signaling components. A) Number of proliferating cells (KI67+) in the SGZ after 1 day and

number of proliferating NBs (KI67+/DCX+/EdU+) after 7 days in the presence of the MCT8 inhibitor Silychristin were determined. B) Slices were exposed to BCH, an inhibitor of L-type amino acid transporters 1/2, and overall proliferation (KI67+ cell numbers), EdU incorporation in general and into proliferating cells after 1 day in culture; in EdU/DCX co-labelled cells after 7 days in culture or in EdU/NEUN double positive cells after 21 days in culture was quantified. C) Slices were cultured in the presence of the deiodinase inhibitor iopanoic acid (IOP). Proliferation at day 1 as well as EdU-incorporation into KI67+ cells at day 1, into DCX+ progenitors at day 7 and into neurons at day 21 is depicted. n=4-6 mice per condition. Group means + SEM are shown.

Fig.S4: Progenitors in the SGZ at two months of age. Related to Fig. 4

Intermediate progenitors and apoptosis were analyzed on perfusion-fixed forebrain cryosections of WT and MCT8 KO mice at 2 months of age. A) Number of cells positive for the intermediate progenitor marker TBR2 (in green) were enumerated. B) Number of apoptotic cells (cleaved caspase-3 positive; green) was compared between WT and MCT8 KO littermates. Hoechst33258 counter-stained nuclei appear blue. n=6 mice per genotype. Group means + SEM are shown.

Fig.S5: Progenitors in the SGZ at six months of age. Related to Fig. 5

At 6 months of age, intermediate progenitor numbers positive for TBR2 (in green/grey; arrowheads) (A) and cleaved caspase-3 positive (in green/grey; arrowheads) apoptotic cells (B) were compared between WT and MCT8 KO mice using perfusion-fixed forebrain cryosections. Hoechst33258 counter-stained nuclei appear blue. n=6 mice per genotype. Group means + SEM are shown.

Fig.S6: Intermediate progenitors in MCT8-NSC KO animals. Related to Fig. 6

Conditional deletion of MCT8 in adult NSCs was achieved by Tamoxifen-induced activation of a *Nestin*-CreERT2 construct at 4 weeks of age and monitored by RFP reporter. A) Adult

generated neurons positive for NEUN (blue) and RFP (magenta) express MCT8 (green; arrows) in control animals, but are devoid of specific MCT8 staining in the MCT8-NSC KO condition when analyzed at 6 months of age. B) In 6 months old animals, relative numbers of TBR2+ (green)/RFP+ (magenta) TAPs in the dentate gyrus were determined and are presented as % of all RFP+ cells. Cell nuclei are labelled with Hoechst33258 and are shown in blue. n=5 mice per genotype. Group means + SEM are shown.

Fig.S7: Cell cycle inhibitor transcript levels in the dentate gyrus. Related to Fig. 7

At 6 months of age, dentate gyri were micro-dissected and cDNA was generated. Transcript expression of *p27kip1* and *p57kip2* was investigated and levels were normalized to that of the stage-specific marker *Dcx*. n=4-5 mice per genotype. Group means + SEM are shown. *, $p < 0.05$, unpaired two-tailed Student's *t* test.

Supplementary experimental procedures:

Tissue preparation for FACS

Dentate gyri were minced with a razor blade on an ice-cold glass plate, collected into Hibernate A and centrifuged for 2 min at 3000 rpm. Generally, all centrifugation steps were conducted at 4 C. Hibernate A was replaced with 1 ml Accutase (Millipore) and tissue was digested for 30 min at 4 C. Subsequently, tissue was pelleted by centrifugation for 2 min at 3000 rpm and re-suspended in 1 ml Hibernate A. To dissociate cells, dentate gyri pieces were triturated on ice using a syringe and needles with decreasing diameter (1.2 mm, 0.8 mm and 0.4 mm). Cells were filtered through a pre-wetted 40 µm cell strainer into a 50 ml tube to remove large debris. Smaller debris was reduced by applying cell solution on top of a three-step Percoll gradient (for recipe see (Guez-Barber et al., 2012)). After initial centrifugation at 4000 rpm for 4 min, the cloudy top layer was aspirated and cells were pelleted by centrifugation at 5000 rpm for 10 min.

Staining procedure for FACS

Following re-suspension in 860 µl ice-cold staining buffer (1x PBS with 0.1% saponine, 1% BSA and 1:25 RNaseOUT) three 20 µl aliquots were taken (for an unstained control and two secondary only controls) and the remaining volume was split into two 400 µl aliquots. The first aliquot was incubated with mouse anti-CR (1:400; Swant #6B3), guinea pig anti-DCX (1:400; Merck Millipore #AB2253) and rabbit anti-TBR2 (1:150; Abcam #ab183991); the second 400 µl cell suspension was incubated with rabbit anti-CB (1:100; Millipore #AB1778), mouse anti-GFAP (1:200;Merck Millipore #MAB360) and chicken anti-NESTIN (1:150; Aves #NES) for 40 min at 4 C. Cells were washed, re-suspended in 400 µl ice-cold staining buffer and incubated with Alexa 488/568/647-coupled secondary antibodies raised in goat (1:400; Invitrogen) for 30 min at 4 C. For secondary only controls, 380 µl staining buffer were added to a 20 µl cell aliquot and cells were incubated with 0.5 µl of a 1:10 mix of all secondary antibodies in staining buffer. After washing, 400 µl ice-cold FACS buffer was added (1x PBS containing 0.5% BSA and 1:50 RNaseOUT) per sample.

Immunofluorescence studies

Coronal forebrain cryosections containing the hippocampus (16 µm) were thaw-mounted on superfrost slides (Thermo Scientific), post-fixed with 4% PFA for 10 min and permeabilized with 0.1% Triton X-100/0.1 M glycine. For EdU detection, sections were blocked in 3% BSA in PBS and Click-iT reaction was performed using the Click-iT® EdU Alexa Fluor® 647 Imaging Kit (Thermo Fisher Scientific) according to the manufacturer's instructions. Subsequently, sections were blocked with MOM reagent (1:40; Vector laboratories) in blocking buffer (PBS containing 10% goat serum and 0.2% Triton X-100) and incubated with the respective primary antibodies in blocking buffer overnight at 4 C. Sections were incubated with Alexa Fluor 488, 568 or 647 labelled secondary antibodies (all 1:1000) in blocking buffer and Hoechst33258 (5 µg/ml), mounted with Fluoromount-G* (Southern Biotech) and analysed using a Leica SP8 confocal microscope.

Organotypic adult hippocampal slices were permeabilized and subjected to Click-iT reaction as above with extended incubation times (2 h blocking in 3% BSA/PBS and 3 h incubation with the Click-iT reaction cocktail). Free-floating slices were blocked overnight in blocking buffer (with 0.5% Triton X-100), incubated for 48 h with primary antibodies and 24 h with secondary antibodies as above in blocking buffer (with 0.5% Triton X-100) and Hoechst33258 (5 µg/ml). Slices were transferred on superfrost slides, mounted and analyzed using a Leica SP8 confocal microscope.

The following primary antibodies were used: mouse anti-CALBINDIN (1:500; Swant), mouse anti-CALRETININ (1:500; Swant #6B3), rabbit anti-cleaved caspase-3 (1:500; Cell Signalling Technology #9664), rabbit anti-DIO3 (1:25; Pierce #PA5-22886), guinea pig anti-DOUBLECORTIN (1:500; Merck Millipore #AB2253), chicken anti-GFAP (1:500; Covance #PCK591P), rabbit anti-KI67 (1:250; Abcam #ab16667), rabbit anti-LAT1 (1:500; TransGenicInc. #KE026), rabbit-anti LAT2 (1:200; immunoGlobe #0142-10), goat anti-MCM2 (1:100; Abcam #ab4461), rabbit anti-MCT8 (1:500; Sigma-Aldrich #HPA003353), rabbit anti-MCT10 (1:50; MyBioSource #MBS2521988), mouse anti-NEUN (1:300; Millipore #MAB377), rabbit anti-P21 (1:250; Abcam #ab188224), rabbit anti-P27 (1:250; Abcam #ab32034), rabbit anti-P57 (1:200; Sigma #P0357), rat anti-SOX2 (1:500; eBioscience #14-9811-80), rabbit anti-TBR2 (1:250; Abcam #ab183991).

qPCR

To exclude the presence of genomic DNA, one sample without reverse transcriptase was included as well. Ten nanograms of cDNA were employed in one qPCR reaction. Three to four samples per population were subjected to the analysis in duplicate. As a housekeeping gene for normalization Glyceraldehyde 3-phosphate dehydrogenase (Gapdh) was used. The annealing temperature was 55°C for all primer pairs. The following primers were chosen to generate the PCR fragments: *Crym* 5'-GTCCAGGCGTACAGTCACTA-3' and 5'-AGCCTCCTGCACTGATGAAC-3'; *Dio1* 5’-CGTGACTCCTGAAGATGATG-3’ and 5’-CCAATGCCTATGGTTCCTAC-3’; *Dio2* 5’-GATGCTCCCAATTCCAGTGT-3’ and 5’-

AGTGAAAGGTGGTCAGGTGG-3'; *Dio3* 5'-ACTAGGCCACATCGATTC-3' and 5'-CAACCTTTGACTTTCTTTGG-3'; *Dcx* 5'-CTACAGAACCAGAACCTTGC-3' and 5'-TCAGAAGAAACAGCGTACAC-3'; *Gapdh* 5'-ATGCCAGTGAGCTTCCCGTC-3' and 5'-CATCACCATCTTCCAGGAGC-3'; *Hes5* 5'-GAAACACAGCAAAGCCTTC-3' and 5'-TGGAAGTGGTAAAGCAGC-3'; *Lat1* 5'-GTCGTTCAGTAGCACAGAG-3' and 5'-CCATCTGTCAGTCCAAGTC-3'; *Lat2* 5'-TTCCCATTATCTACCTGCTG-3' and 5'-GAACTCCTGTCAGCATAATG-3'; *Mct8* 5'-TGCCGAATCACAGCCACCAC-3' and 5'-GGCGAATGAGCAGCCACAAC-3'; *Mct10* 5'-GGCCGCATTGCTGACTATTT-3' and 5'-CAATGGGCGCCATGATAGA-3'; *Ncor* 5'-GCAAAGTCGTTATCCTTCAC-3' and 5'-ATGCCTGGCTAACTTCAATA-3'; *Neun* 5'- ATCGGAACCATGTGAAACC-3' and 5'-GTAGCCGGGTGTACGCCAGA-3'; *Oatp1c1* 5'-GCTGTGGAAAACTCAAGGTG-3' and 5'-GGAAGGGATCTCAAACCTTC-3'; *p27kip1* 5'-ACATATCGCTGACTCCATTG-3' and 5'-GCAGATGGTTTAAGAGTGCC-3'; *p57kip2* 5'-CCCCCACTTCATTAGATTGC-3' and 5'-TTATTTAGAACCTGGCGGAC-3'; *Prox1* 5'-TCCGACATCTCACCTTATTC-3' and 5'-TCCGAGAAGTAGGTCTTCAG-3'; *Smrt* 5'-CAACAAGAAACTCAACACCC-3' and 5'-CTTCTACAGGTCATAAGGCC-3'; *Trα1* 5'-GGCAGTTATCTTGTCCCTTT-3' and 5'-CAAGTAAGCACAGACGACTA-3'; *Trα2* 5'-CCGCTTCCTCCACATGAAAG-3' and 5'-CAGCTCTGTCCCTTCTCTCC-3'; *Trβ1* 5'-TGAAGAATGAGCAGACTTCC-3' and 5'-CTTGAGATGCTCTGATCGTT-3'; *Trβ2* 5'-GTAGTTACCCTGGAAACCTG-3' and 5'-GAAAAGGCTTTTCTTCAGGG-3'.

Quantification

Marker positive cells (cleaved caspase-3, CB, CR, DCX, EdU, GFAP, KI67, NEUN, RFP, SOX2, and TBR2) in the SGZ were counted and normalized to the length of the SGZ using ImageJ (NIH). To determine the number of GFAP+/SOX2+ NSCs, broader z-stacks were imaged, and only cells that extended a single GFAP+ process into the granule cell layer were counted. In RFP-labelling experiments, numbers of marker positive/RFP+ cells were counted

and expressed as percentage of all RFP+ cells so as to account for inter-animal variability. In all histo-morphological studies, 5 to 6 images from 3 to 4 sections per animal were quantified.

Relative integrated densities of P21, P27 and P57 immunofluorescence signals were calculated by encircling the nuclei and dividing the value by the analyzed area. Background intensity was measured in the same way by encircling a nucleus-free area in the hilus region, and subtracted from the signals calculated above. 5 pictures per animal and a total of 25 cells (WT vs. MCT8 Ko at P60), 16-25 cells (WT vs. MCT8 KO at P180), and 5-19 cells (control vs. MCT8-NSC KO at P180) per cell type were analyzed.

To evaluate *p27* mRNA levels in the DCX+ population, we reasoned that *Dcx* mRNA levels per cell would be the same in Wt and ko animals even if the NB numbers were different. We therefore calculated the *p27* mRNA/*Dcx* mRNA ratio from dissected hippocampal tissue using RT-PCR protocols described above.

RT-PCR

RNA was isolated, amplified and transcribed into cDNA as above. 12.5 µg cDNA were employed in a standard PCR using an annealing temperature of 55 C and respective qPCR primer pairs. PCR products were visualized on a 3% agarose gel using a G:Box system (Syngene).
